# Transient Inhibition of FGFR2b-Ligands Signaling Leads to Irreversible Loss of Cellular β-Catenin Organization and Signaling in AER during Mouse Limb Development

**DOI:** 10.1371/journal.pone.0076248

**Published:** 2013-10-22

**Authors:** Soula Danopoulos, Sara Parsa, Denise Al Alam, Reza Tabatabai, Sheryl Baptista, Caterina Tiozzo, Gianni Carraro, Matthew Wheeler, Guillermo Barreto, Thomas Braun, Xiaokun Li, Mohammad K. Hajihosseini, Saverio Bellusci

**Affiliations:** 1 Keck School of Medicine, University of Southern California, Los Angeles, California, United States of America; 2 Developmental Biology and Regenerative Medicine Program, Saban Research Institute of Childrens Hospital Los Angeles, Los Angeles, California, United States of America; 3 Life Science Division, Lawrence Berkeley National Laboratory, Berkeley, California, United States of America; 4 Nassau University Medical Center, Pediatric Department, New York, New York, United States of America; 5 Department of Internal Medicine II, University of Giessen Lung Center and Member of the German Lung Research Center (DZL), Giessen, Germany; 6 Departement of Cardiac Development and Remodelling, Max-Planck Institute for Heart and Lung Research and Member of the DZL, Bad Nauheim, Germany; 7 Max-Planck-Institute for Heart and Lung Research, LOEWE Research Group Lung Cancer Epigenetic, Bad Nauheim, Germany; 8 School of Pharmacy, Wenzhou Medical College, Wenzhou, China; 9 School of Biological Sciences, University of East Anglia (UEA), Norwich, Norfolk, United Kingdom; Stockholm University, Sweden

## Abstract

The vertebrate limbs develop through coordinated series of inductive, growth and patterning events. Fibroblast Growth Factor receptor 2b (FGFR2b) signaling controls the induction of the Apical Ectodermal Ridge (AER) but its putative roles in limb outgrowth and patterning, as well as in AER morphology and cell behavior have remained unclear. We have investigated these roles through graded and reversible expression of soluble dominant-negative FGFR2b molecules at various times during mouse limb development, using a doxycycline/transactivator/tet(O)-responsive system. Transient attenuation (≤24 hours) of FGFR2b-ligands signaling at E8.5, prior to limb bud induction, leads mostly to the loss or truncation of proximal skeletal elements with less severe impact on distal elements. Attenuation from E9.5 onwards, however, has an irreversible effect on the stability of the AER, resulting in a progressive loss of distal limb skeletal elements. The primary consequences of FGFR2b-ligands attenuation is a transient loss of cell adhesion and down-regulation of P63, β1-integrin and E-cadherin, and a permanent loss of cellular β-catenin organization and WNT signaling within the AER. Combined, these effects lead to the progressive transformation of the AER cells from pluristratified to squamous epithelial-like cells within 24 hours of doxycycline administration. These findings show that FGFR2b-ligands signaling has critical stage-specific roles in maintaining the AER during limb development.

## Introduction

Congenital anomalies of the limb occur between 1 in 500 to 1 in 1000 live human births [1]. They are often the result of a complex interaction of multiple minor genetic abnormalities with environmental risk factors. Intrauterine disruption of limb development by exposure of the fetus to teratogenes, usually between the third and eighth week of gestation, can also per se lead to anomalies of the limb. The precise molecular and cellular bases of these anomalies are still largely unknown and require a better understanding of normal limb development.

The growth and patterning of vertebrate limbs is regulated by epithelial-mesenchymal cell interactions, involving a diverse array of signaling pathways. In mouse, limb development along the body flank is initiated with the formation of forelimb buds at embryonic day 9.5 (E9.5) followed 12 hours later by hindlimb buds. The early limb bud is composed of mesenchymal cells derived from the lateral plate mesoderm, covered by ectoderm cells, a subset of which converge at the dorso-ventral border of limb buds to form a pluristratified structure termed the Apical Ectodermal Ridge (AER) [2]. As simple as the anatomy of the limb bud appears, it is composed of many signaling zones that control the temporal and spatial specification of the mesenchymal progenitors in the limb. These signaling zones determine the three different axes of the mature limb: proximal-distal (PD), anterior-posterior (AP) and dorso-ventral (DV). Along the PD axis, the skeletal elements of mature limbs can be divided into three domains: the proximal stylopod (humerus/femur), the zeugopod (radius/tibia and ulna/fibula) and the distal autopod (carpal/tarsal, metacarpal/metatarsal, phalanges).

The AER is the earliest signaling domain to be induced during limb bud formation. It is formed by the action of FGF10, produced by lateral plate mesoderm, on Fibroblast Growth Factor Receptor 2b (FGFR2b), expressed by ectoderm cells [3]–[4]. FGF10 also induces the expression of *Fgf8* in the AER, which in turn acts on the underlying mesenchymal cells located in the putative “undifferentiated zone” (UZ) thus allowing the amplification of the different skeletal progenitors of the limb [5] (for review see [6]).

Using a non targeted transgenic approach to express constitutively and ubiquitously a soluble form of FGFR2b acting as a dominant negative receptor, it was reported that mutant embryos at E18.5 display a large variety of defects including complete limb agenesis or agenesis of the hindlimb associated with truncated forelimb with missing autopod [7]. Further investigations of the role of FGFR2b signaling in limb development have confirmed the previous results and produced some interesting but conflicting results. Using a *Msx2-Cre* driver line to abrogate FGFR2b function in the AER after forelimb AER formation but before hindlimb AER formation, Lu et al. [8] reported decreased *Fgf8* expression and loss of AER, resulting in the complete loss of the autopod skeletal elements in the forelimb as well as hindlimb agenesis. However, no changes in cell proliferation or cell death were observed in the distal mesenchyme of the forelimb bud. Yu and Ornitz [9] used the same transgenic approach to discover reduced mesenchymal cell proliferation at E10.5–E11 but not E10.0, with no impact on cell death. Analysis of *Hoxa13* expression, a marker of autopod progenitors, showed that *Msx2-Cre*-mediated targeting of *Fgfr2*, reduces the pool of autopod progenitors [8]. Since developmental events occur at a rapid pace in the developing limbs, one drawback of using a Cre line to delete *Fgfr2* expression is that it will not abrogate the activity of the endogenous FGFR2b protein present prior to the complete inactivation of the conditional *Fgfr2* allele. Therefore, upon Cre activation, the precise analysis of the resulting phenotype and the associated primary defects are limited by the presence of a residual FGFR2 activity which persistence will depend on its stability. In addition, as the deletion of *Fgfr2* in the AER is irreversible, it is difficult to investigate whether restoring FGFR2 signaling in the AER is sufficient to re-induce AER formation.

Here, we report the use of a novel approach to robustly target signaling induced by FGFR2b-ligands interaction within a relatively narrow window of time at desired developmental time points, in order to investigate its role/s at later stages on limb outgrowth and patterning, as well as on AER morphology and cell behavior. In this model, soluble dominant-negative FGFR2b molecules are generated by administration of doxycycline to mice harboring a dox/transactivator tet(O)-responsive transgene. We find that transient FGFR2b-ligands signaling inhibition leads to permanent disorganization of β-catenin, a cellular component critical for both cell adhesion and signaling. In harmony with this result, restoration of FGFR2b-ligands signaling after a 24-hour inhibition period is not sufficient to trigger de novo AER formation. We also demonstrate that interruption of FGFR2b-ligands signaling affects distinct elements of the limb skeleton in a time and dose-dependent manner.

## Results

### FGFR2b-ligands signaling controls progressive limb growth along the proximal-distal axis

We have previously reported that we could use a double transgenic (DTG) [*R26^rtTA+/−^;TetOsFgfr2b/+*] (also called [*R26^rtTA/+^;Tg/+*]) in vivo system to inducibly and reversibly attenuate FGFR2b-ligands signaling in the post-natal mouse [10]. The *Rosa26^rtTA/+^* line allows the ubiquitous expression of the transactivator rtTA. The *tetOsFgfr2b/+* line contains a construct allowing the expression of a secreted fusion protein composed of the extracellular part of FGFR2b fused with the heavy chain domain of immunoglobulin. Upon exposure to doxycycline, the transactivator rtTA is activated and will induce the expression of this fusion protein, which will be transported outside the cell. We therefore expect this fusion protein to act in a non-cell autonomous fashion as a decoy receptor for FGFR2b-ligands.

**Figure 1:**
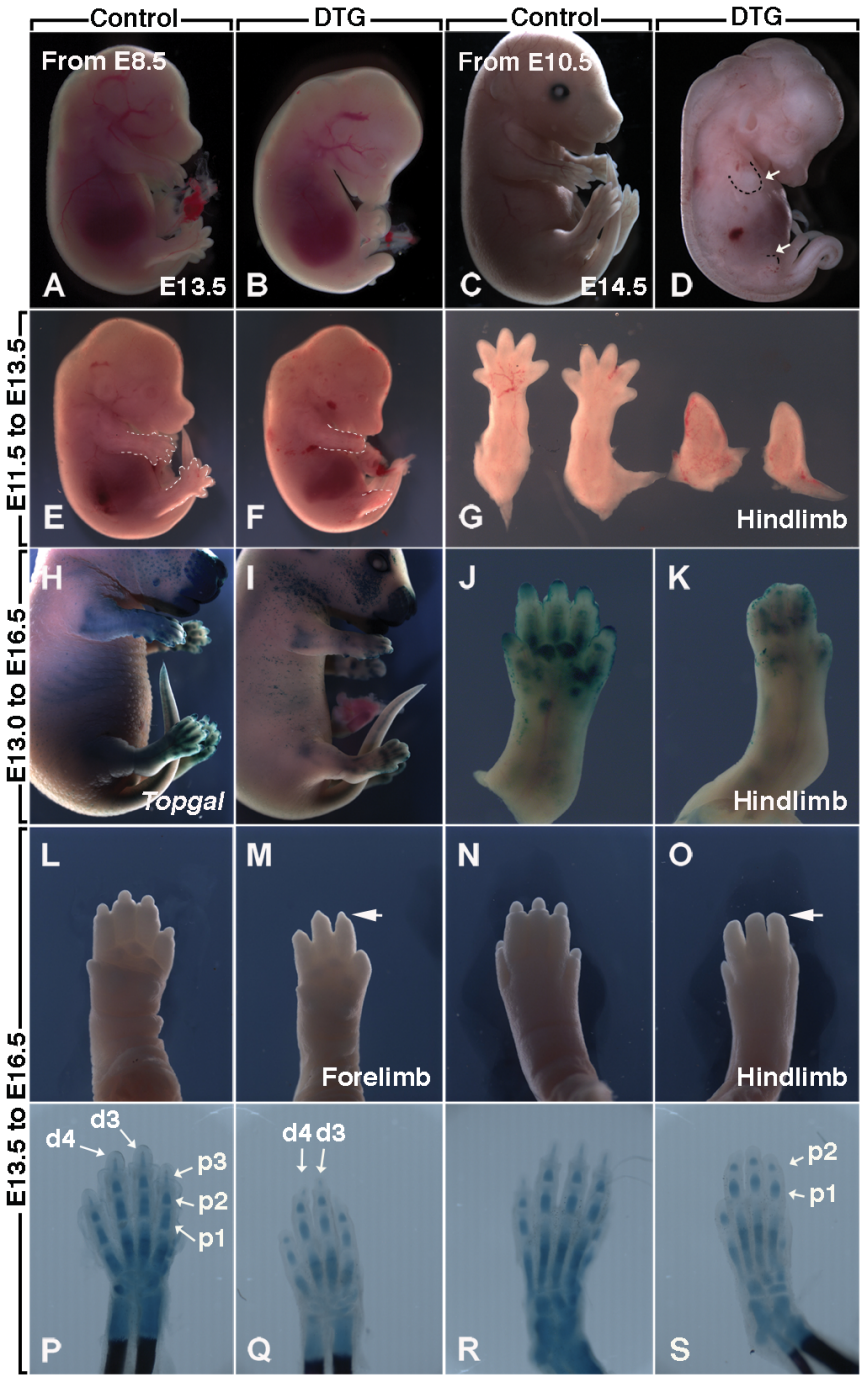
Signaling induced by FGFR2b-ligands interactioncontrols progressive limb growth along the proximal-distal axis. Pregnant females carrying [*R26^rtTA/+^;Tg/+*] double transgenic (DTG) embryos and single transgenic [*R26^rtTA/+^ or Tg/+*] control embryos were treated continuously with Doxycycline food starting at different developmental stages; (**A,B**) Treatment at E8.5, before limb induction: loss of both hindlimbs and forelimbs in E13.5 DTG embryos. (**C,D**) Treatment at E10.5, after limb bud induction: Formation of rudimentary forelimbs and almost complete absence of hindlimbs in E14.5 DTG embryos. (**E–F**) Treatment at E11.5: Absence of autopod in both hindlimbs and forelimbs of E13.5 DTG embryos. (**G**) Dissected hindlimbs in DTG and controls shown in (E,F). (**H–I**) Treatment at E13.0: control (H) and DTG (I) embryos at E16. Note that the *Topgal* allele was introduced in DTG and control embryos to visualize the extent of mesenchymal condensation in the limb. (J,K) Dissected left hindlimbs from embryos shown in H and I displaying failure of separation of the digits in DTG hindlimb. (**L–O**) Treatment at E13.5: truncation of the digits in both forelimbs and hindlimbs. (**P–S**) Alcian blue/alizarin red staining indicates the reduction in the size of the P3 phalange in the forelimb and complete loss of the P3 phalange in the hindlimb of DTG embryos treated from E13.5 to E16.5. d, digits; p, phalanges.

We first aimed to validate the use of our double transgenic mice in the context of limb development. Pregnant females carrying both DTG heterozygous and control embryos were placed on Doxycycline containing (Dox) food between E8.5 and E13.5, corresponding to the pre-induction, induction and post-induction stages of mouse limb buds. DTG embryos (n = 5 out of 5, the phenotypes described thereafter are always 100% penetrant) exposed to Dox food from E8.5 (pre-induction) to E13.5 failed to generate limbs (Fig. 1A,B). Skeletal preparation of these embryos sacrificed at E18.5 indicates a complete failure of limb development phenocopying *Fgfr2b* null embryos [5] (data not shown). By contrast, exposure at E10.5 (early limb bud), 24 hours after forelimb induction and only 12 hours after hindlimb induction, resulted in shorter forelimbs and very rudimentary hindlimbs in DTG embryos (n = 15, Fig. 1C, D). Dox treatment from E11.5 (late limb bud) to E13.5 led to the absence of autopod in both forelimb and hindlimb in the DTG embryos (n = 11, Fig. 1E–G). Dox treatment from E13.0 to E16.5 led to defective separation of the digits (Fig. 1H–K). To better visualize the impact on cartilage condensations, DTG embryos carrying an additional *Topgal* allele [11] were used (Fig. 1H–K). Dox treatment from E13.5 to E16.5 led to shortening of all five digits in both forelimbs and hindlimbs (ratio digit length in DTG versus control HL: 78.5±7.3%, FL: 84.3±6.4%) with digits 1 and 5 being more severely affected (n = 20). Skeleton staining was carried out to better visualize this phenotype and examine the affected cartilage condensations, only to discover shorter phalanx 1 and 2 as well as the specific absence of the third phalanx in the hindlimb (Fig. 1P–S) (ratio phalanx length in DTG versus control limbs P1: HL: 88.5±3.1%, FL: 86.8±9.1%, P2: HL: 72±8.5%, FL: 77±16.7%, P3: HL: 0%, FL: 47±0.7%). In conclusion, the brachydactyly phenotype is associated with defective proximal-distal growth of the digits with completely missing phalange 3 in the HL. This phenotype is reminiscent of the digit defects (ranging from aplasia to hypoplasia), which most often involved the thumbs in patients with hypomorphic *FGF10* mutations [12]. Interestingly, the forelimbs are slightly less affected than the hindlimbs, indicating that the asynchrony in mouse fore- and hindlimb development continues well into E13.5. No obvious limb defects were observed in DTG heterozygous embryos exposed to Dox food from E14 onwards (n = 17, data not shown). However, we cannot exclude a role for FGFR2b-ligands signaling in limb development beyond E14, which, if any, is likely to involve ossification rather than specification and patterning of the skeleton. The postnatal functionality of limbs in Dox-treated DTG embryos is difficult to analyze because they all die at birth from abnormal lung development (Al Alam and Bellusci, unpublished observation).

Taken together, these data validate the use of DTG mice to attenuate specifically FGFR2b-ligands signaling in the developing limbs and show that the role of FGFR2b-ligands is not confined to the induction and autopod initiation phases, as previously described (E8.5 to E13.5) [5], [8], [9]. Rather, FGFR2b signaling continues to play a role in digit outgrowth.

### Impact of transient FGFR2b-ligands attenuation before and after limb bud induction on the formation of the skeletal elements along the Proximal/Distal axis

**Figure 2:**
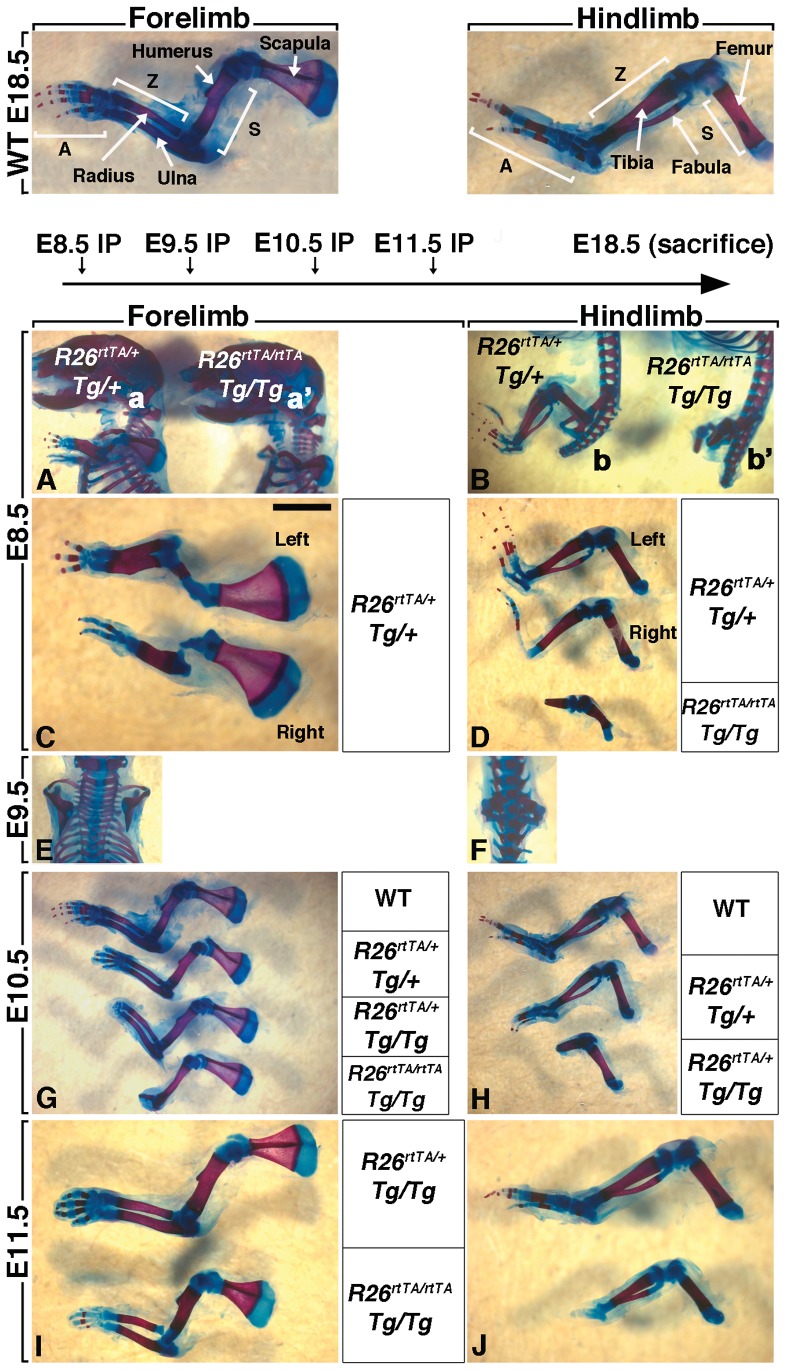
Transient attenuation of FGFR2b-ligands signaling, before and after limb bud induction, affects different skeletal elements along the A/P axis. Alcian blue/alizarin red staining of embryos was used to visualize the bone versus cartilage respectively. Images of E18.5 embryos which were exposed to Dox at E8.5 (**A–D**), E9.5 (**E–F**), E10.5 (**G,H**) and E11.5 (**I–J**). Hindlimbs demonstrate more drastic defects than forelimbs. Also, homozygous vs. heterozygous embryos and right vs. left limbs are differently affected.

To examine the impact of transient FGFR2b-ligands signaling attenuation on the formation of the limb skeleton, pregnant females carrying DTG and control embryos were given a single Dox-IP dose at E8.5, E9.5, E10.5 and E11.5 and their embryos were collected and analyzed at E18.5. We also used different allelic combination of *R26^rtTA^* and *Tg* to determine the impact of the gene dosage on the severity of the limb phenotype. Double heterozygous transgenic embryos ([*R26^rtTA/+^;Tg/+*]) exposed to Dox-IP at E8.5 (1–1.5 days before forelimb and hindlimb induction, respectively) displayed shorter forelimbs but quasi-normal hindlimbs (n = 7, Fig. 2A and B, left embryo). This result supports our claim that inducible inhibition of FGFR2b-ligands signaling is indeed reversible, as the sustained blockade of FGFR2b-ligands signaling between E8.5 and E13.5 led to limb agenesis (Fig. 1A,B). However, double homozygous transgenic embryos [*R26^rtTA/rtTA^;Tg/Tg*] did not produce forelimbs and had severely truncated hindlimbs (n = 10, Fig. 2A and B, right embryo). Double heterozygotes (Fig. 2C) showed normal scapula but reduced stylopod (humerus) and zeugopod (radius and ulna) (n = 7). Interestingly, the right limbs were more severely affected, with a near absence of right humerus and shortened femur, and the complete absence of right ulna and fibula. Furthermore, the autopodal digits had formed in both fore and hindimbs, but these were fewer on the right side. By comparison, the double homozygous DTGs had much shorter femur and no elements beyond a rudimentary tibia. Unexpectedly, double homozygous DTG embryos exposed to Dox-IP at E9.5, at the time of forelimb induction but 12 hours before hindlimb formation, displayed a complete absence of both forelimbs and hindlimbs (n = 13, Fig. 2E,F) suggesting that FGFR2b-ligands attenuation at E9.5 leads to irreversible loss of the AER.

We next examined the consequences of inhibiting FGFR2b-ligands signaling at E10.5, 24 and 12 hours after forelimb and hindlimb induction, respectively. Mutant forelimbs displayed a progressive loss of distal skeletal elements as a function of the genotype (Fig. 2G). [*R26^rtTA/+^; Tg/+*] embryos (n = 11) displayed only a truncation of the digits, whilst in [*R26^rtTA/+^; Tg/Tg*] embryos (n = 9) the autopod was completely absent, as previously shown in Fig. 1. In [*R26^rtTA/rtTA^; Tg/Tg*] embryos (n = 14), the entire autopod and the bulk of zeugopod failed to develop. The corresponding phenotypes were more severe in the hindlimbs (Fig. 2H). For example, [*R26^rtTA/rtTA^; Tg/Tg*] embryos failed to generate any hindlimbs (n = 14, data not shown) and the [*R26^rtTA/+^; Tg/Tg*] embryos displayed an aborted zeugopod but a normal stylopod (n = 9). These data are consistent with an irreversible loss of the AER from a single Dox-IP injection at E10.5. The level of inhibition of FGFR2b-ligands signaling at this time point and its subsequent impact on the amplification of the mesenchymal progenitors that will later form skeletal elements in the zygopod and autopod correlates with the genotype. Our results also indicate that at E10.5, the mesenchymal progenitors that will later contribute to the stylopod (humerus and femur in fore and hind-limb, respectively) do not require FGFR2b-ligands signaling for their specification and amplification.

Finally, the inhibition of FGFR2b-ligands signaling at E11.5, 48 and 36 hours after forelimb and hindlimb induction, respectively led only to digit defects in the forelimb of both [*R26^rtTA/+^; Tg/Tg*] (n = 13) and [*R26^rtTA/rtTA^; Tg/Tg*] (n = 16) embryos (Fig. 2I and Fig. S1). The [*R26^rtTA/rtTA^; Tg/Tg*] hindlimbs (n = 13) exhibited an almost complete absence of autopod while in the [*R26^rtTA/+^; Tg/Tg*] hindlimb (n = 16) it was normal (Fig. 2J). Interestingly the truncation of the autopod appears to form with a distal to proximal gradient suggesting that the AER did not recover from transient FGFR2b-ligands inhibition at E11.5.

These results indicate that at all of the post-limb bud induction time-points studied - E9.5, E10.5 and E11.5 - the AER does not recover from transient attenuation of FGFR2b-ligands signaling. Hence, we next aimed to define the role of FGFR2b-ligands signaling on AER morphology and cell behavior.

### Dynamics of soluble *Fgfr2b* expression and activity in vivo after single Dox-IP administration at E11

**Figure 3:**
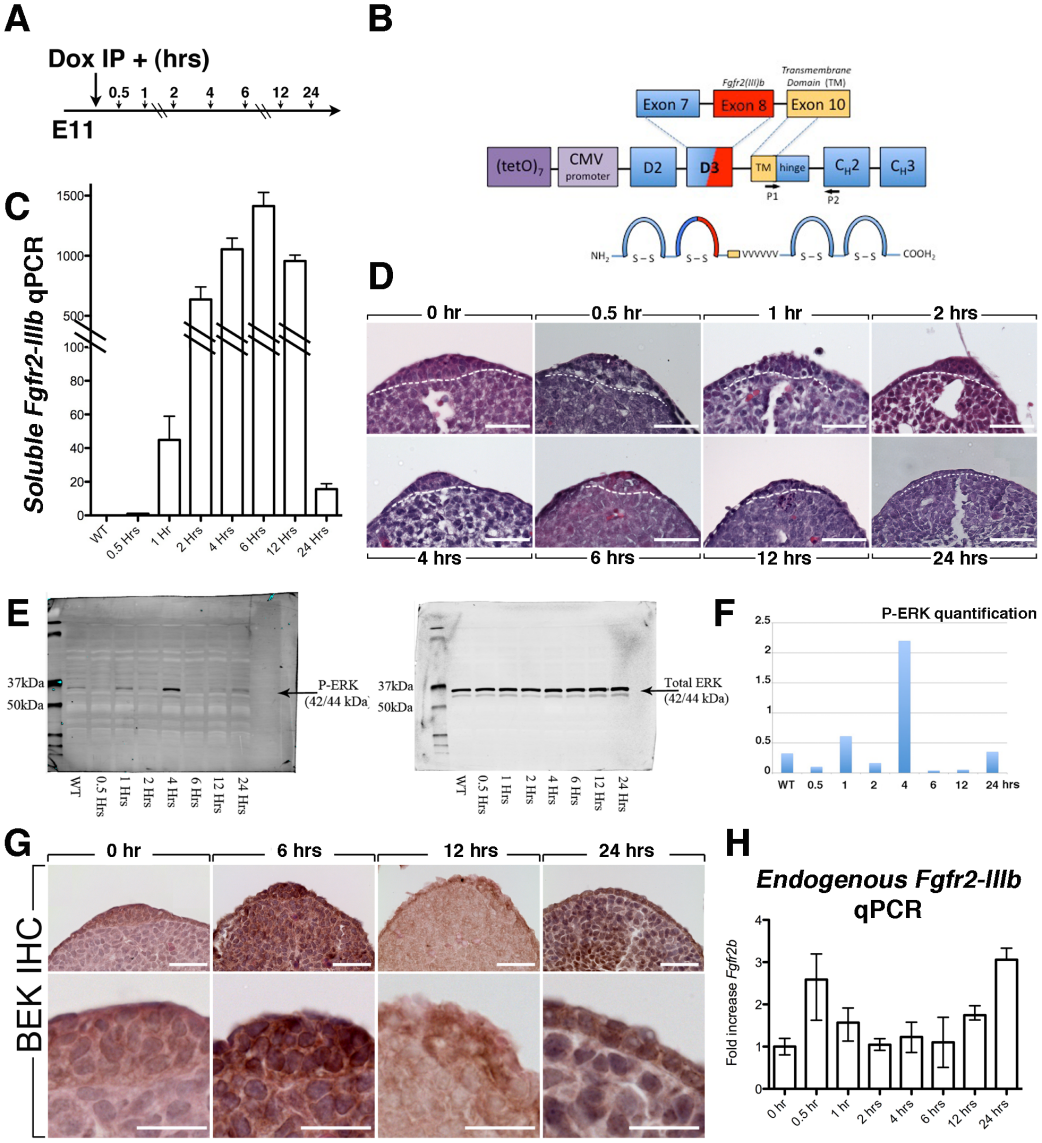
Dynamics of soluble *Fgfr2b* expression and impact on AER maintenance after a single Dox-IP injection. (**A**) Embryos were collected at 0.5, 1,2,4,6,12 and 24 hrs after Dox IP at E11. (**B**) Schematic of the *soluble Fgfr2b* structure indicating the position of the specific primers P1 and P2 used to detect *sFgfr2b* expression. (**C**) Quantification of *soluble Fgfr2b* by qRT-PCR indicating a peak of expression at 6 hrs and a steep decrease at 24 hrs. (**D**) Analysis of the AER at these different time points showing a progressive diseappearance of the AER. (**E**) Western blot from whole E11 embryos exposed to Dox at different time points. Significant decrease in P-ERK levels is observed after 4 hrs. (**F**) Quantification of the P-ERK/Total ERK ratio at the different time points. (**G**) BEK (FGFR2) expression by IHC indicating that FGFR2 is still expressed in the rudimentary AER at 24 hrs post Dox-IP. (**H**) qRT-PCR for endogenous *Fgfr2b* expression supporting the IHC results. Scale bar D: 50 µm; G-upper panels: 50 µm; G-lower panels: 25 µm.

The initial experiments shown in Fig. 1 relied on sufficient uptake of Dox-containing food over a period of days by pregnant females carrying DTG heterozygous embryos. However, the fate of mesenchymal progenitors is determined within narrow time windows between E9.5 to E11.5. Therefore, to be able to pinpoint the role/s of FGFR2b-ligands signaling in different developmental processes, we refined our experimental approach by delivering a single dose of Doxycycline (1.5 mg/kg/body weight) intraperitoneally (Dox-IP) at different time points (Fig. 2). In order to determine the time frame during which we can characterize the primary consequences of FGFR2b attenuation, we aimed to define the level of soluble *Fgfr2b* expression at different time points after a single Dox-IP administration in pregnant females carrying E11 [*R26^rtTA/rtTA^; Tg/Tg*] homozygous embryos (Fig. 3A). We isolated the right forelimbs of DTG embryos at 0.5, 1, 2, 4, 6, 12 and 24 hours after Dox-IP and measured soluble *Fgfr2b* expression in each limb independently (n = 3) by qRT-PCR using specific primers which do not recognize the *endogenous Fgfr2b* transcripts (Fig. 3B). Our results indicate that *sFgfr2b* expression is detected as early as 30 minutes after Dox IP and reaches a maximum of expression at 6 hours followed by a steep decline at 24 hours. Interestingly, the levels of *sFgfr2b* expression at 24 hours in double homozygous embryos are still 10 times higher compared to the ones observed at 30 minutes (Fig. 3C). Histological analysis of the corresponding left forelimbs (n = 3) showed progressive AER disappearance (Fig. 3D) with no visible AER at 24 hours post Dox-IP while an ectodermal thickening is still observed in the corresponding time-matched controls (data not shown). Inhibition of FGFR2b-ligands activity was investigated by western blot using p-ERK antibodies on whole embryo extracts (n = 1 per well) collected at different time points after Dox-IP at E11. The western blot was repeated 2 more times using independent embryos. We consistently found (n = 3) that during the first 4 hours after Dox-IP, p-ERK is increased suggesting the involvement of compensatory mechanisms upon FGFR2b signaling attenuation. However, between 4 and 24 hours, p-ERK expression is almost completely extinct only to be found re-induced at 24 hours (Fig. 3E.F). Interestingly, endogenous FGFR2 expression in the epithelium investigated using BEK Antibodies (recognizing an epitope localized in the C-terminal cytoplasmic domain of FGFR2 which is common to the epithelial and mesenchymal isoforms of FGFR2) is maintained in the AER/ectoderm of the forelimb over time after Dox-IP administration (Fig. 3G, n = 5). This result was confirmed by qRT-PCR using specific primers for endogenous *Fgfr2b* (Fig. 3H, n = 4).

### Loss of *Fgf8* expression in DTG AER after Dox-IP injection

**Figure 4:**
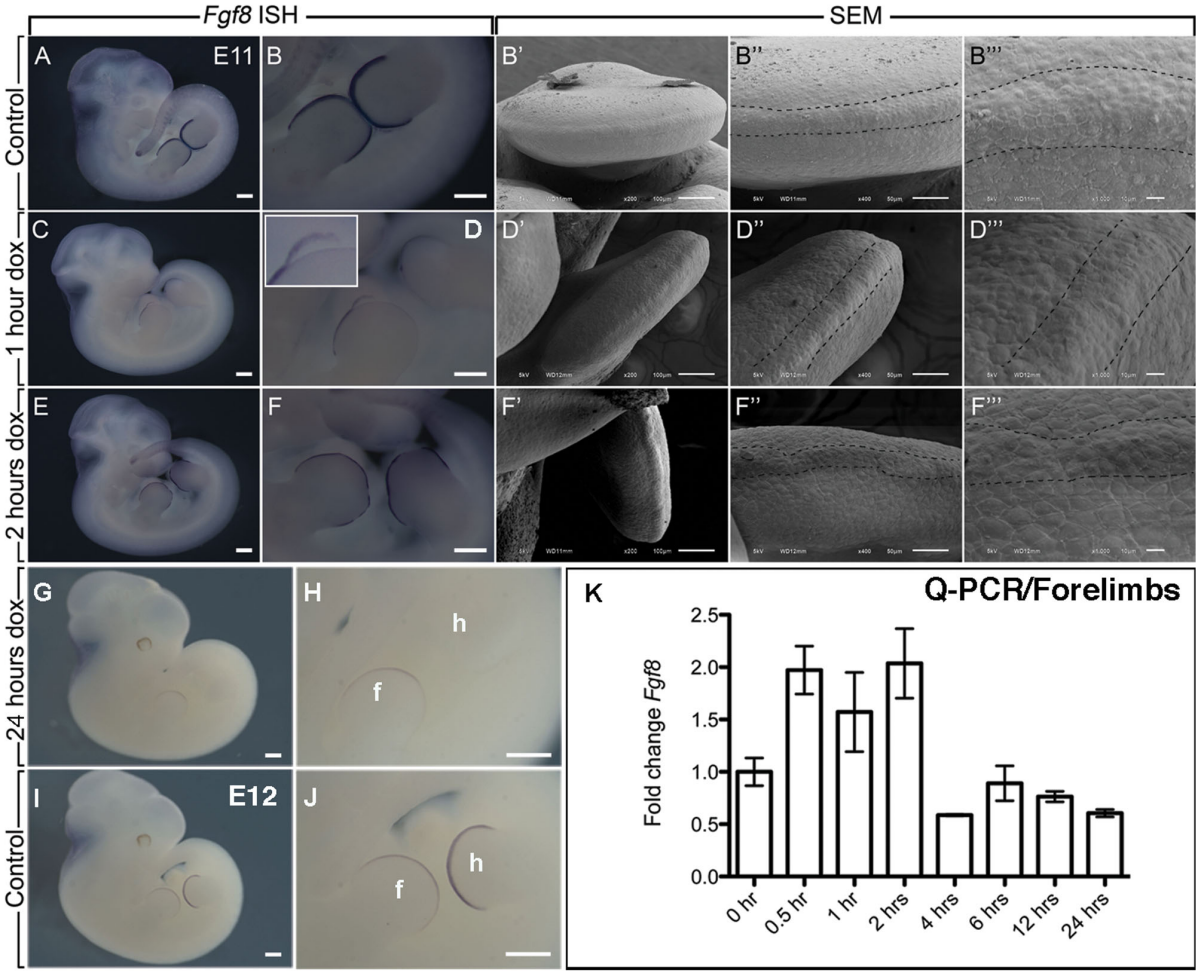
Expression of *Fgf8* in the AER following Dox-IP. (**A–F**) WMISH for *Fgf8* in control (**A,B**), *Rosa26^rtTA/rtTA^; Tg/Tg* embryo after 1 hour Dox-IP injection (**C,D**) and 2 hours after Dox-IP injection (**E,F**). B,D,F are high magnification of A,C,E. (C,D) *Fgf8-AER* expression is decreased at 1 hour after Dox-IP injection. (inset in D) Close up examination of the limb shows that the AER is detaching. (E,F) *Fgf8-AER* is significantly expressed 2 hours after Dox-IP injection. However, note that the expression at 2 hours is still lower than the one observed in the control and still exhibits gaps in *Fgf8* expression indicating defective AER at this time point. (**B′–B′″,D′–D′″,F′–F′″**) SEM images of control (**B′–B″**), 1 hour Dox-IP (**D′–D″**) and 2 hours Dox-IP (**F′–F″**) embryos showing that the AER is still present at 1 and 2 hrs after Dox-IP. (**G,H**) *Fgf8* expression at 24 hrs post Dox-IP showing complete absence of *Fgf8* expression in the hindlimb and a residual expression in the forelimb. (K) Quantification of *Fgf8* expression by qRT-PCR in the forelimbs at different times post Dox-IP. Note that *Fgf8* expression is still present at 1 hr post Dox-IP supporting the hypothesis that the lack of *Fgf8* expression at 1 Dox-IP by WMISH is due to the physical loss of the AER. Scale bar A,C,E,G,I: 500 µm; B,D,F,H,J: 300 µm; B′,D′,F′: 100 µm; B″,D″,F″: 50 µm; B′″,D′″,F′″: 10 µm.

To test AER functionality, we checked the expression of *Fgf8* by WMISH at t = 0 (control), 1 hour and 2 hours post Dox-IP delivery to E11 *R26^rtTA/rtTA^; Tg/Tg* homozygous embryos. *Fgf8* expression is strongly expressed in the AER of control limbs (Fig. 4A,B). Moreover, the AER can be easily visualized as an elevated ridge at the interface between the dorsal and ventral limb by Scanning Electron Microscopy (SEM) (Fig. 4B′–B″). Only one hour post Dox-IP injection, DTG AERs showed abnormal *Fgf8* expression (Fig. 4C, n = 8) with defects ranging from absence of *Fgf8* expression along the limb (n = 3 out of 8 DTG embryos) to gaps in *Fgf8* expression in the AER (n = 5 out of 8 DTG embryos). However, close up examination of the forelimbs indicated that in our experimental conditions, the AER, expressing *Fgf8*, was detaching (inset in Fig. 4D). Interestingly, SEM indicated that the AER is still present in the limb 1 hour after Dox-IP (Fig. 4D–D″) suggesting that the lack of *Fgf8* expression at 1 hour post Dox-IP is due to the physical detachment of the AER as the likely consequence of decreased cell adhesion rather than decreased *Fgf8* RNA level. We propose that such decrease in cell adhesion is particularly apparent following enzymatic treatment during the WMISH procedure. Supporting a transient change in the cell adhesion status at 1 hour post Dox-IP, *Fgf8*-AER expression was almost normal at 2 hours after Dox-IP, bar a few localized domains (Fig. 4E–F). Like for the 1 hour post Dox-IP time point, SEM analysis at 2 hours post Dox-IP indicated the presence of a structurally defined AER (Fig. 4F′–F″). Interestingly, after 24 hours post Dox-IP, *Fgf8* expression is almost completely absent in the hindlimb but still present in the forelimb (Fig. 4G,H) compared to the corresponding E12 control limbs (Fig. 4I,J). We also measured directly *Fgf8* expression in dissected FL by qPCR over time (Fig. 4K).

Our results indicated increased *Fgf8* expression within the first 2 hours following Dox-IP delivery, confirming that the absence or decreased expression of *Fgf8* after 1 hour Dox-IP observed by WMISH is due to the detachment of the AER and not to the loss of *Fgf8* expression *per se*. From 4 hours onwards, *Fgf8* expression is reduced confirming the progressive loss of a functional AER.

### Cellular and Molecular defects in the AER appear shortly after attenuation of FGFR2b-ligands signaling

**Figure 5:**
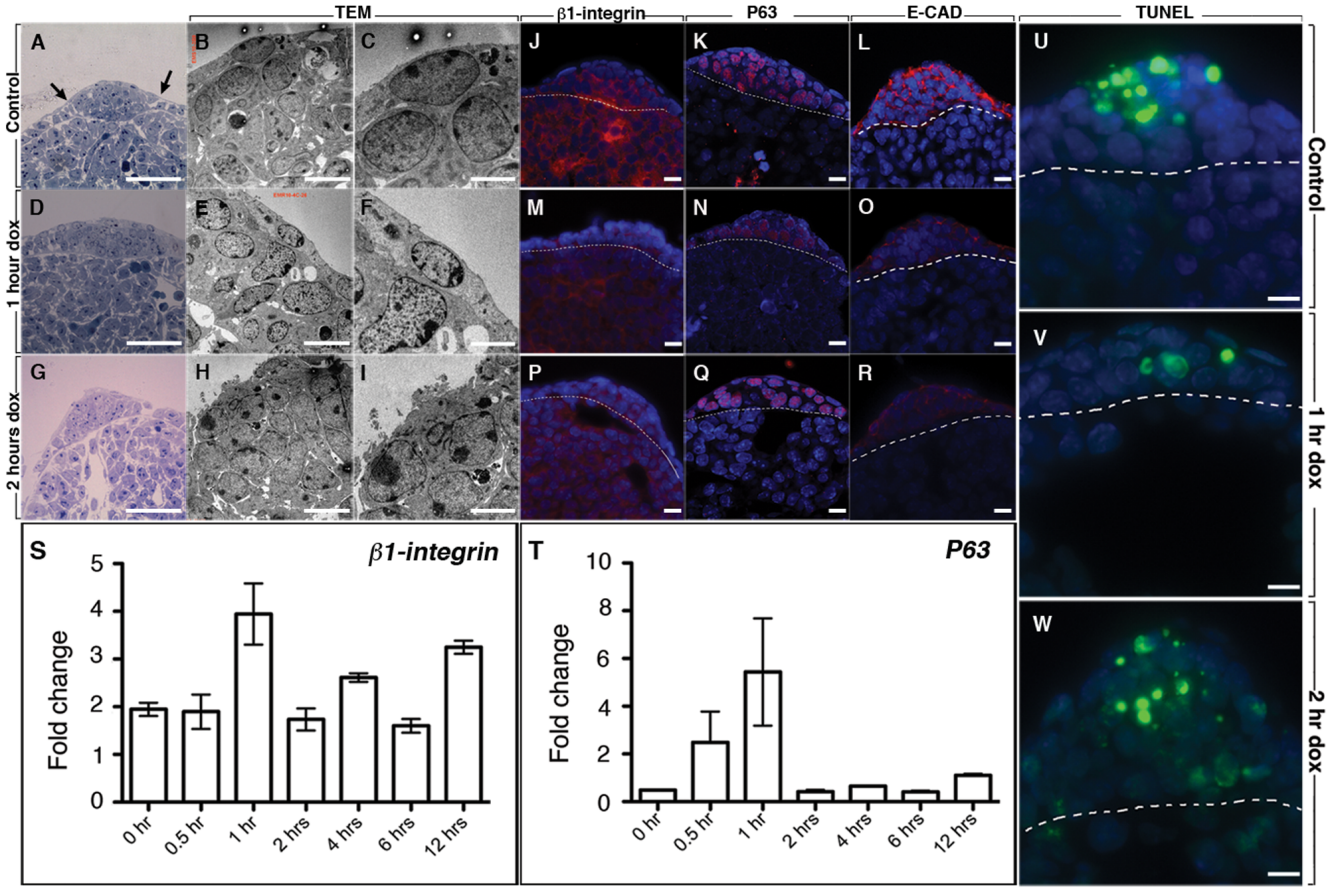
Cell adhesion and cell death are reduced in the AER after attenuation of FGFR2b-ligands signaling. Histology (**A, D, G**) and TEM (**B, C, E, F, H, I**) images of the AER at E11, E11+ 1 hr and E11+ 2 hrs post Dox-IP. (D) Note that at 1 hr the AER is spreading and no longer a compact pseudostratified epithelium like in the control (A). SEM analysis does not indicate major changes except for irregularly shaped nuclei. (G–I) after 2 hrs Dox-IP, the AER seemingly reformed as a compact structure (G) but SEM analysis indicated that the most superficial layer, the periderm is missing. (**J–R**) Cell-cell adhesion was tested by IF for β1-integrin (J,M,P), P63 (K,N,Q) and E-Cadherin (L,O,R) expression. β1-integrin expression is reduced in DTG-AER 1 and 2 hours after Dox-IP (**M, P**) in comparison to the control AER at E11 (**J**). P63 expression is reduced in DTG-AER 1 hours after Dox-IP (**N**) compared to the control AER at E11 (**K**). No significant difference is observed at 2 hours after Dox-IP (**Q**) compared to the control AER. E-cadherin expression is reduced in DTG-AER 1 and 2 hours after Dox-IP (**O,R**) in comparison to the control AER at E11 (**L**). (**U–W**) TUNEL staining for control AER at E10.5 (**U**), DTG-AER 1 hour after Dox-IP (**V**) and 2 hours after Dox-IP (**W**) demonstrate reduction in cell death at 1 hour after Dox-IP in the DTG-AER. (**S and T**) Quantification by q-PCR of *β1-integrin* and *P63* expression. Scale bar A,D,G: 25 µm; B,E,H: 5 µm; C,F,I: 2 µm; J–R: 20 µm; U–W: 20 µm.

To further study the cellular and molecular defects in the AER of double homozygous DTG [*R26^rtTA/rtTA^; Tg/Tg*] embryos, we injected pregnant females with a single dose of Dox-IP at E11 and examined the cellular structure of the AER 1 and 2 hours later by Transmission Electron Microscopy (TEM). Analysis of semi-thin sections showed that wild type AER (n = 4) is normally composed of three to four layers of polystratified epithelial cells with smooth and round nuclei (Fig. 5A), and covered by periderm, a thin monolayer of elongated squamous like cells (Fig. 5B,C). The border between the AER and the adjacent squamous epithelium was also clearly visible (black arrows in Fig. 5A). However, one hour after Dox-IP (n = 5), this border is no longer visible, the epithelial cells in the AER are no longer compactly arranged at the rim and some of the nuclei became irregular in shape (Fig. 5D–F). Two hours after Dox-IP (n = 4), the appearance of the superficial peridermal cells is abnormal while the cells in the AER appear again more compacted (Fig. 5H–I).

The cellular changes in the AER could result from the dispersion of the AER cells into the adjacent squamous epithelium following changes in their cell adhesion status or amplified cell death. To investigate whether these are due to a reduction in the cohesive properties of the AER cells, we first examined the levels of β1-integrin, the transcription factor P63 and E-cadherin, which are known to regulate cell adhesion [13]–[16]. Levels of β1-integrin showed a marked decrease within 1 and 2 hours post Dox-IP injection in DTG-AER (n = 4 for each time point) (Fig. 5M,P) compared to the control-AER (n = 5) (Fig. 5J). This decrease also occurred in the underlying mesenchyme. A transient reduction in P63 was observed 1-hour post Dox-IP injection (n = 3) (Fig. 5K,N) but this was partially restored within the following hour (n = 4) (Fig. 5Q vs. K). E-cadherin expression was decreased at 1 and 2 hours post Dox-IP (n = 5 for each time point) (Fig. 5O, R) compared to the E11 control AER (n = 4) (Fig. 5L) confirming the cell adhesion defects upon FGFR2b-ligands signaling attenuation. We also measured the expression of β1-integrin and P63 by qRT-PCR in FL at different times after Dox-IP (Fig. 5S,T) (n = 3 for each time point). Our results indicate a compensatory increase at the RNA level of the expression of these two genes during the first 0.5–1 hour of Dox treatment.

Examination of cell death levels by TUNEL staining showed a transient but significant reduction in AER cell death, 1 hour post Dox-IP injection (n = 5), compared to the control (n = 5) (Fig. 5U,V). Apoptosis levels were subsequently increased in the AER 2-hours after Dox-IP injection (n = 5)(Fig. 5W).

Based on these observations, we propose that upon inhibition of FGFR2b-ligands signaling at E11, the multistratified and tightly-compacted epithelial cells in the AER lose their cell-cell and/or cell-matrix adhesion to resemble their neighboring squamous epithelial cells. Also, apoptosis, which is a feature of a functional AER, is decreased as an immediate response to attenuation of FGFR2b-ligands signaling. Altogether, our results indicate that the AER does not recover at the structural, cellular and molecular level from a single dose of Dox-IP.

### Transient loss of AER function affects mesenchymal cell proliferation but not cell death

**Figure 6:**
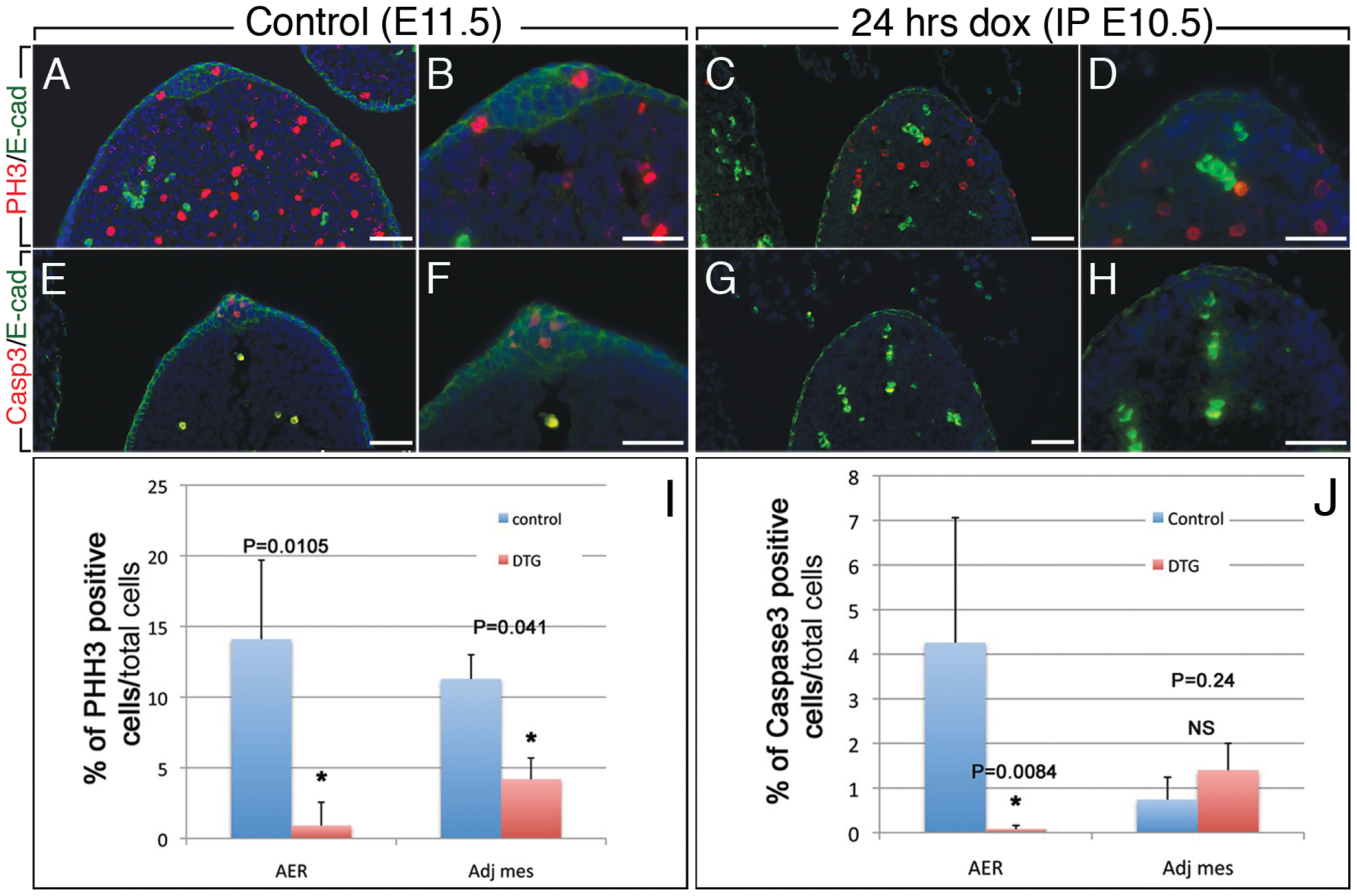
FGFR2b-ligands signaling controls cell proliferation in the mesenchyme of the limb bud. (**A–D**) Phosphohistone H3 and E-cadherin double IF staining in control E11.5 (**A,B**) and DTG [*R26^rtTA/rtTA^; Tg/Tg*] (**C,D**) limb bud exposed to Dox-IP at E10.5 and analyzed at E11.5 demonstrate significant reduction in cell proliferation of both the AER and the adjacent mesenchyme. (**E–H**) Caspase 3 IF staining for cell death in control limb bud at E11.5 (**E,F**) and DTG (**G,H**) limb buds exposed to Dox-IP at E10.5 and analyzed at E11.5 display significant decrease in apoptosis of the rudimentary AER, there is no change in the apoptosis of the adjacent mesenchyme. (Adj mesenchyme = Adjacent mesenchyme). (**I**) Quantification of PHH3 positive cells. (**J**) Quantification of caspase 3 positive cells. Bars represent the mean ± s.e.m. of at least 5 independent samples of each. Mann-whitney non-parametric test was performed. *p≤0.05. Scale bar A–H: 50 µm.

We next investigated the impact of FGFR2b-ligands attenuation on cell proliferation and apoptosis in the AER and the adjacent mesenchyme by carrying out double immunolabeling for E-cadherin in combination with either Phosphohistone H3 or cleaved Caspase 3, on DTG homozygous embryos (n = 5) exposed to a single Dox-IP injection at E10.5 and examined 24 hours later. E11.5 limbs were used as controls (n = 5). We found a significant decrease in cell proliferation in both the AER and the adjacent mesenchyme (Fig. 6A,B vs. C,D,I). As suggested from Fig 2G,H, the AER did not recover from FGFR2b-ligands attenuation at E10.5. In order to quantify the defects in the AER, we considered the single layer of epithelium, stained positive for E-cadherin at the very tip of the limb, as the rudimentary AER in the mutants and examined proliferation and cell death in this structure. In the controls, 14.1% of the cells in the AER stained positive for PHH3 while only 0.9% were positive in the AER of the DTG (p = 0.0103; Fig. 6I; n = 5). Similarly, in the adjacent mesenchyme, 11.2% of cells were proliferating in the controls versus 4.2% in the DTG (p = 0.041; Fig. 6I, n = 5). As expected and due to the quasi-absence of AER, a significant decrease in cell death was observed in the DTG AER (Fig. 6G, H) as compared to control AER (Fig. 6E, F; 0.08%); 4.26% versus 0.08%, respectively (p = 0.0084; Fig. 6J, n = 5). No significant change in cell death was observed in the DTG mesenchyme (Fig. 6G, H) as compared to the controls (Fig. 6E, F): 1.4% in the DTG versus 0.8% in the control (p = 0.24; Fig. 6J, n = 5).

### The vascular system in the limb is conserved following FGFR2b-ligands signaling attenuation

Abnormal limb development has recently been connected with impaired vascular formation [17]. We therefore examined the integrity of the vascular system in the E11 limbs at 6 hours post Dox-IP by anti-PECAM immunofluorescence labeling, and validated our results by qRT-PCR, quantifying the expression of *Pecam* and *Vegfa*. Our results indicated no significant differences in PECAM expression between control (Fig. S2A,B) and experimental limbs (Fig. S2C,D). In both cases, endothelial cells were found to be abundant in the limb mesenchyme. Interestingly, PECAM expression is not enriched at the level of the AER suggesting that, unlike other organs such as the lung, the endothelial cells do not interact directly with the AER for its maintenance via the secretion of angiocrine factors. The quantification of *Pecam* expression by qRT-PCR (Fig. S2E) indicated a slight increase in *Pecam* expression in the mutant limbs over the time period considered (0.5–24 hours) while *Vegfa* expression did not change significantly (Fig. S2F). Overall, we did not observe major impairment of the vascular system upon inhibition of FGFR2b-ligands signaling.

### Impact of FGFR2b-ligands attenuation on mesenchymal progenitor differentiation

**Figure 7:**
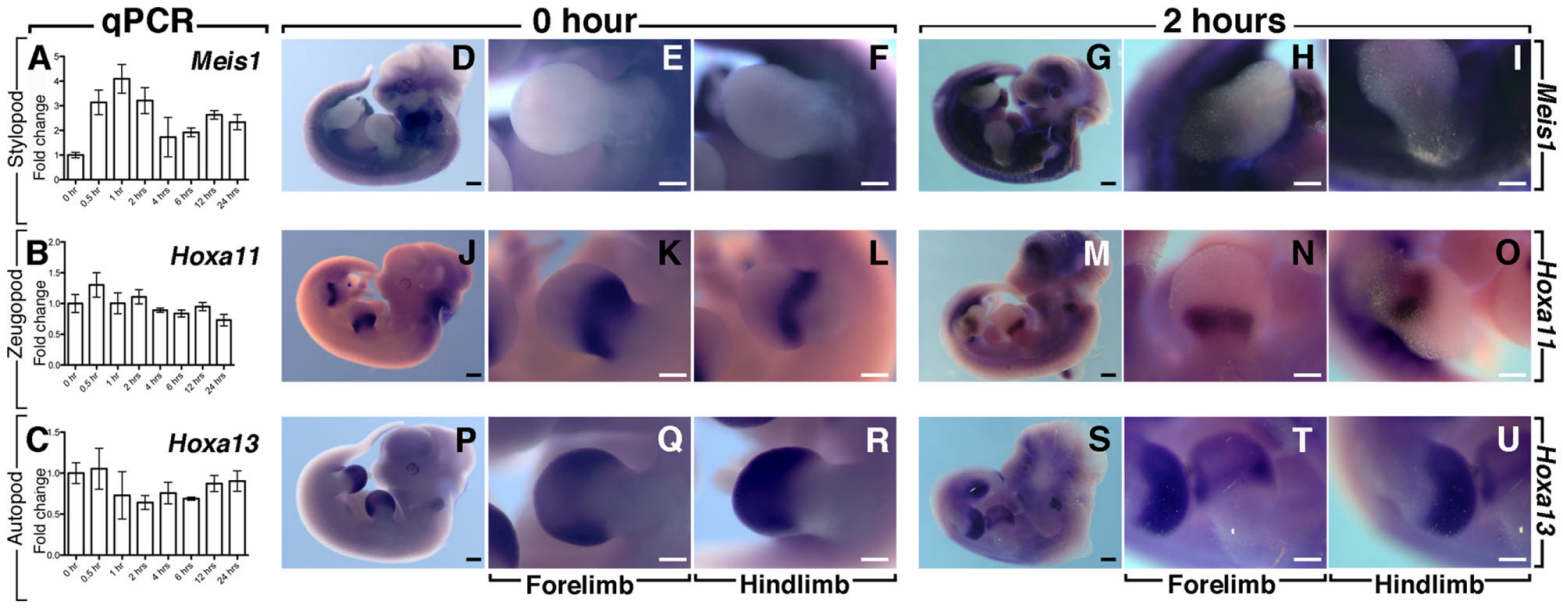
Fate of the mesenchymal progenitors upon FGFR2b-ligands inactivation. (**A–C**) Quantification by qRT-PCR of *Meis1*, *Hoxa11* and *Hoxa13* expression in the developing forelimb at different time-points after Dox injection at E11. (**D–U**) WMISH at 0 hr (D–F; J–L; P–R) and 2 hours Dox-IP (G–I; M–O; S–U) for *Meis1* (D–I), *Hoxa11* (J–O) and *Hoxa13* (P–U). Note the increase in the expression of proximal/stylopod progenitor marker *Meis1* at the expense of the distal/autopod marker *Hoxa13*. Scale bars: D,J,P,G,M,S: 500 µm; E,F,K,L,Q,R,H,I,N,O,T,U: 300 µm.

The transcription factors *Meis1*, *Hoxa11* and *Hoxa13* have been previously reported as specific markers for the stylopod, zeugopod and autopod [8]. We measured the level and pattern of expression of these genes by qRT-PCR at different times after Dox-IP treatment (0, 0.5, 1, 2, 4, 6, 12, 24 hrs; 3 independent limbs were used for each time point) and by WMISH 2 hours after Dox-IP (n = 3; Fig. 7), respectively in double homozygous embryos. We found that the expression of the stylopod marker *Meis1* is drastically increased by qRT-PCR upon FGFR2b-ligands attenuation (Fig. 7A). This increase was confirmed by WMISH (Fig. 7G–I vs. D–F). The expression of the zeugopod marker *Hoxa11* showed a decrease by WMISH (Fig. 7M–O vs. J–L) but this was not found significant by qRT-PCR (Fig. 7B). The autopod marker *Hoxa13* was decreased between 1 and 6 hrs post Dox-IP (Fig. 7C). This last result was also confirmed by WMISH (Fig. 7S–U vs. P–R). Overall these results support that FGFR2b-ligands signaling acts indirectly to control the formation of the mesenchymal progenitors in the autopod domain. However, as the overall pattern of gene expression by WMISH at E11 plus 2 hours is not drastically perturbed, this result indicates that the observed distal limb truncations upon inhibition of FGFR2b-ligands interaction is due to cell death or/and reduced proliferation but not to proximalization (change in cell fate) of the mesenchymal progenitors.

### Loss of canonical WNT signaling in the AER after attenuation of FGFR2b-ligands signaling

**Figure 8:**
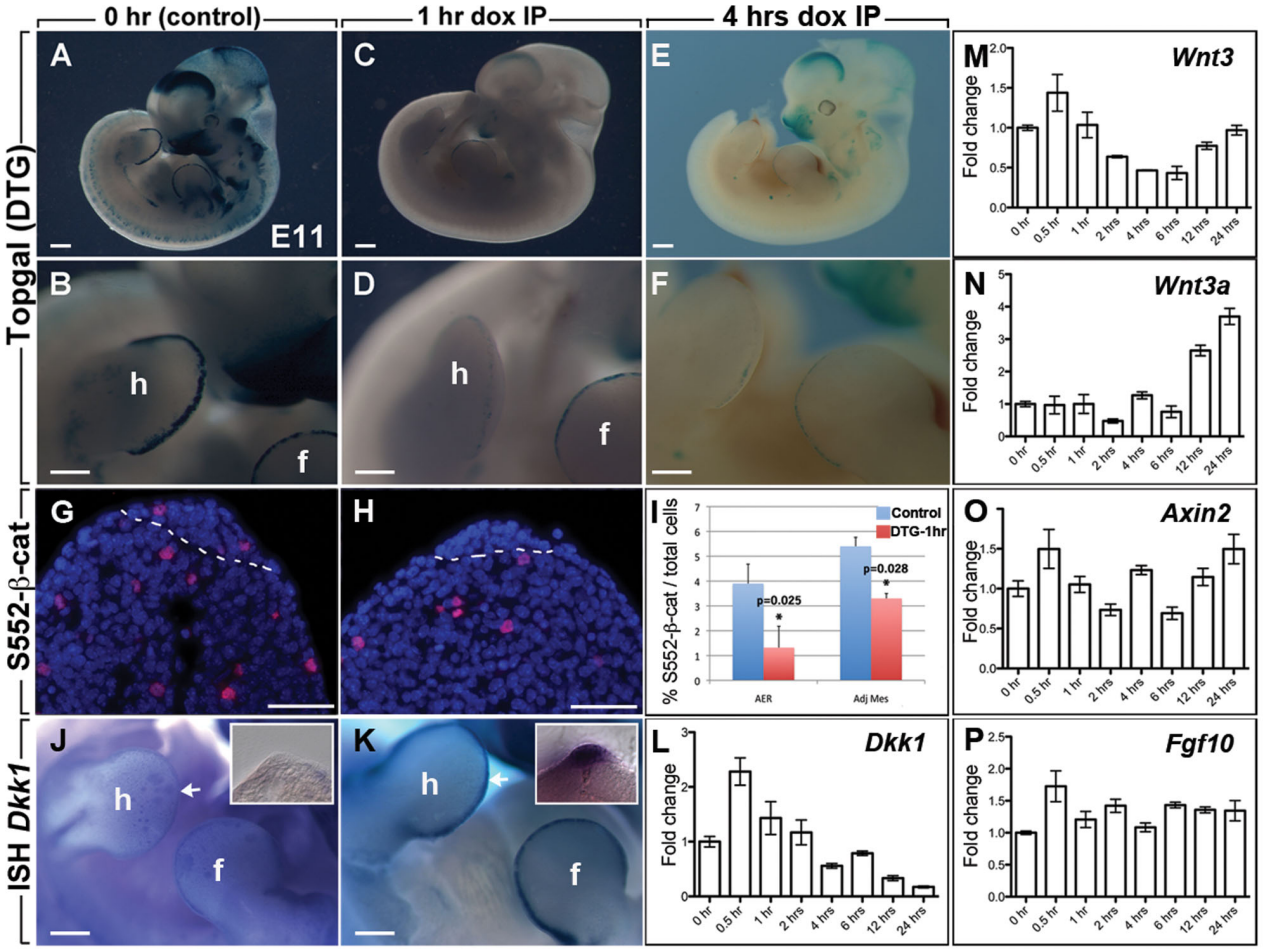
Loss of canonical WNT signaling in the AER after attenuation of FGFR2b-ligands signaling. (**A,B**) *Topgal*, a WNT signaling reporter shows strong expression in the AER of both fore- and hind-limbs of the control embryos at E11. (**C,F**) *Topgal* expression is mostly lost in the AER of DTG embryos 1 hour (C,D) and 4 hrs (E,F) after Dox-IP injection at E11. (**G–H**) IF for the activated form of β-catenin in control (G) and DTG one hour after Dox-IP at E11 (H). Note the strong reduction in β-catenin positive cells in the AER and in the adjacent mesenchyme confirming the *Topgal* results. (**I**) Quantification of G and H. Bars represent the mean ± s.e.m. of at least 5 independent samples of each. Mann-Whitney non-parametric test was performed; *p≤0.05. (**J,K**) WMISH for *Dkk1* indicating robust up-regulation of the WNT inhibitor 1 hour after Dox-IP (K) compared to the control limbs (J). Insets in J and K are corresponding vibratome cross sections through the limb focusing on the AER. (B,D,F are high magnification of A,C,E respectively). (**L–P**) Quantification by qRT-PCR of *Dkk1* (L), *Wnt3* (M), *Wnt3a* (N), *Axin2* (O) and *Fgf10* (P) at different time points after single Dox-IP injection at E11. h, hindimb; f, forelimb. Scale bar A,C: 170 µm; B,D,H,I: 50 µm; E,F: 50 µm.

Females carrying triple heterozygous transgenic embryos (DTG [*R26^rtTA/+^; Tg/+*], as well as *Topgal*, a reporter for WNT signaling [11] that is strongly expressed in the AER [18], were injected with Dox-IP at E11 and embryos were harvested one hour later and stained with X-gal substrate. As evidenced in Fig. 8A–B, control embryos (n = 5) showed a robust and specific expression of LacZ (Topgal) in the AER of their fore- and hindlimb buds. A few Topgal-positive cells were found in the adjacent dorsal and ventral ectoderm. By contrast, in DTG herozygous embryos (n = 7) the level of Topgal expression was drastically reduced in the AER (Fig. 8C,D), more so in the hindlimb compared to the forelimb buds (Fig. 7D). Similar results were observed 2 hours (n = 5, Fig. 8E,F) as well as 12 and 24 hours after Dox-IP (Fig. S3). These results indicate that FGFR2b-ligands attenuation has a rapid impact on WNT signaling in the AER. To seek further evidence that WNT signaling is reduced in AER of Dox-IP treated ([*R26^rtTA/rtTA^; Tg/Tg*]) homozygous embryos, and assess its impact, we measured the level of Serine 552 phosphorylation on β-catenin protein (Fig. 8G,H). A significant decrease in the number of S552-β-catenin positive cells was noted both in the AER and the mesenchyme upon FGFR2b-ligands attenuation (3.9% vs. 1.3% in the AER and 5.4% vs. 3.3% in the mesenchyme, p = 0.025 and 0.028, respectively, n = 4 for each genotype). To determine the potential cause of this decrease in canonical WNT signaling, we examined *Dkk1* expression by WMISH, a known WNT signaling inhibitor, only to find a significant up-regulation in the AER of homozygous [*R26^rtTA/rtTA^; Tg/Tg*] (n = 7) embryos when compared to controls (n = 6) (Fig. 8J,K). We also investigated the expression of *Dkk1*, *Wnt3*, *Wnt3a*, *Axin2* and *Fgf10* expression by qRT-PCR. *Dkk1* expression is increased at 0.5–2 hours confirming the WMISH result but then decreases progressively to very low levels in the following 4–24 hours Dox-IP period (Fig. 8L) making it unlikely that the sustained inhibition of WNT signaling following FGFR2b-ligands attenuation is due to this known WNT signaling inhibitor. We also found that *Wnt3*, a ligand expressed in the epithelium and critical for AER maintenance, is decreased between 2 and 12 hours only to reach normal levels at 24 hours (Fig. 8M). The expression of *Wnt3a*, expressed in the mesenchyme in mice progressively increases over time (Fig. 8N). Interestingly, levels of *Axin2* expression, used as a read out for global WNT signaling in the epithelium and mesenchyme fluctuated overtime (Fig. 8O), a likely reflection of the compensatory mechanisms at work following FGFR2b inactivation. Finally, the expression of *Fgf10* is quite stable for the time frame considered (Fig. 8P). To further determine which WNT signaling component were being altered, we performed a limited WNT signaling pathway qRT-PCR array in forelimbs of one E11 DTG embryos following 6 hours Dox-IP compared to 0 hr. We identified potential genes of interest that are up-regulated such as *Frzb, Pitx2, and Wnt8a* or down-regulated such as *Wnt2b*, *Wnt3a*, *Wnt3*, *Wnt7a*, *Wnt7b*, *Wnt16*, *Fzd4*, *Fzd8*, *Fzd9*, *Wif1*, and *Wisp1*. The expression of these genes was then carefully assessed by qRT-PCRs using individual forelimbs of four independent embryos in the control (0 hr) and experimental (6 hr Dox-IP) group. The changes in all the above genes were statistically validated as shown in Table S2. Interestingly, *Wnt3*, *Wnt7a* and *Wnt7b* are all ligands expressed in the ectoderm [19] and could therefore account for the decreased WNT-AER signaling. In addition, other *Wnt* ligands are such as *Wnt2b*, *Wnt16* as well as the *Wnt* receptors *Fzd4*, *8*, *9* and the *Wnt* negative regulators *Wif1* and *Wisp1* are expressed mostly in the proximal mesenchyme during early limb development (see Table S2 and [19] suggesting that transient inhibition of FGFR2b-ligands signaling has a profound impact on WNT signaling arising from both the epithelium and the mesenchymal compartment.

### FGF10 directly activates β-catenin signaling

To confirm that FGF10 directly activates WNT/β-catenin, cells transfected with the β-catenin reporter TOPFLASH, were treated with FGF10, WNT3A or LiCl for 1 and 6 h (Fig. S4, n = 3 for each time point and condition). As expected, stimulation of cells with known WNT/β-catenin agonists (WNT3A and LiCl) led to an increase in activity. FGF10 treatment resulted in a 1.6 fold increase in β-catenin activity after 1 hr. Interestingly, this increased activity appeared transient since we did not observe an increase in β-catenin activity after 6 hrs of treatment. Stimulation of cells with both FGF10 and WNT3A together did not lead to a further increase in β-catenin whilst cells transfected with the control plasmid TOPFLASH were unresponsive to all growth factors (Data not shown).

### Attenuation of FGFR2b-ligands signaling leads to cellular disorganization of β-catenin

**Figure 9:**
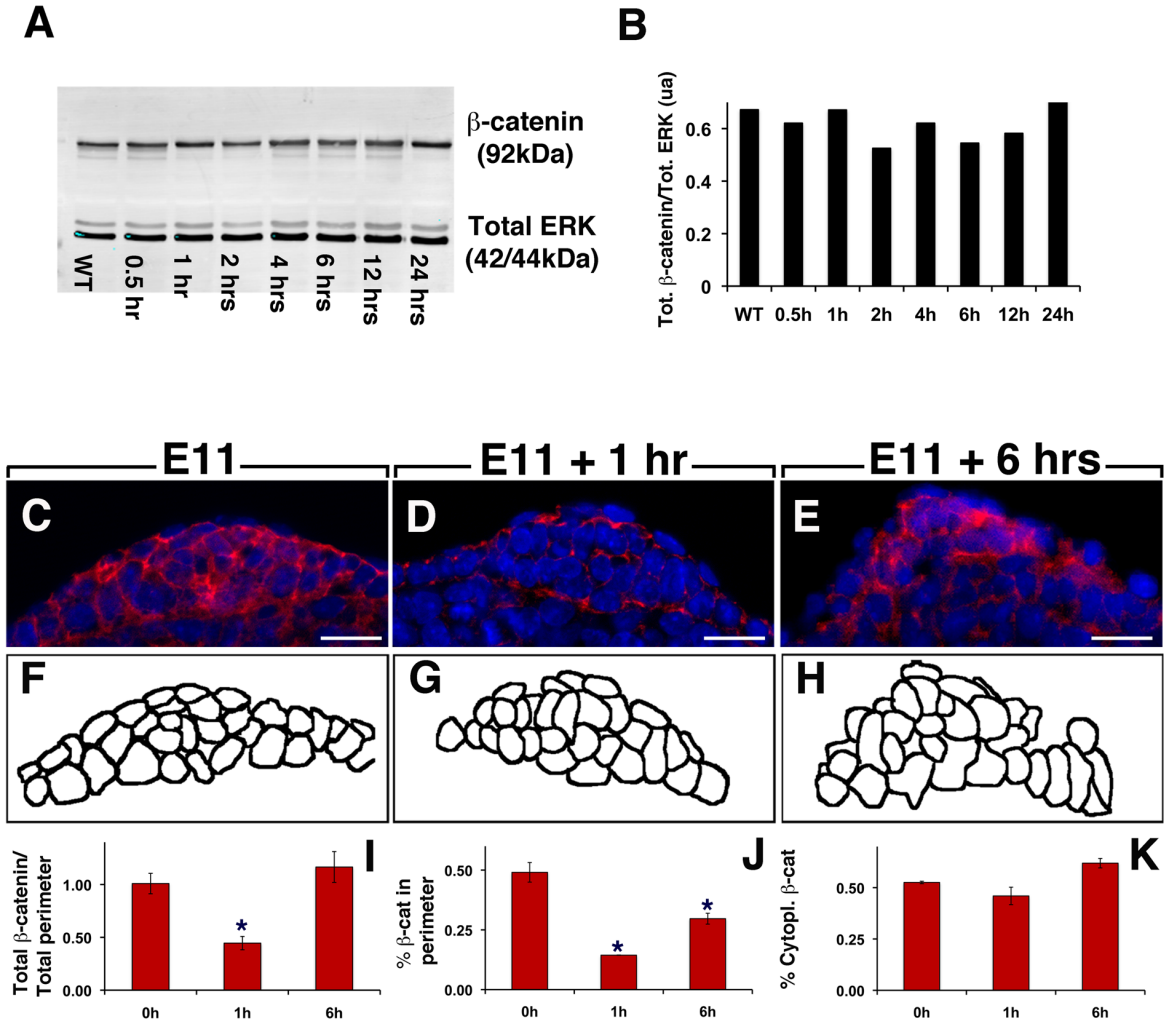
Attenuation of FGFR2b-ligands signaling leads to cellular disorganization of β-catenin. (**A**) Western blot of total β-catenin using protein extract from whole embryo isolated at different time-points after a single Dox-IP injection at E11. (**B**) Quantification of the western blot shown in A. (**C–E**) IF for β-catenin in the AER of E11 forelimbs at 0 hr, (C), 1 hr (D) and 6 hrs (E) after Dox-IP injection. (**F–H**) Schematic representation of the individual cells corresponding to the images shown in (C–E). (**I–K**) Quantification of total β-catenin in the AER (I), total β-catenin at the plasma membrane (J) and total β-catenin in the cytoplasmic compartment (K). Scale bars: A,C,E: 500 µm; B,D,F,F,J: 300 µm; C–E: 20 µm.

The loss of WNT signaling in the AER led us to further examine the cellular localization of β-catenin. It has been reported previously that inactivation of β-catenin in the AER leads to the loss of this structure [20]. Examination of total β-catenin by western blot (n = 3 using independent embryos for each time point) in the whole embryo between 0 and 24 hrs (n = 1 for each time point) after Dox-IP showed that total β-catenin is not drastically affected over this time period (Fig. 9A,B) even though a trend towards a decrease in global β-catenin expression was observed after 2 hrs Dox-IP. Careful examination of β-catenin expression by IF at the level of the AER at 0, 1 and 6 hrs after Dox-IP (n = 3 for each time point) revealed significant changes in cellular localization of β-catenin (Fig. 9C–E). Quantification of the IF signal in the AER between 1 hr Dox-IP and 0 hr (n = 3 for each time point) indicated decrease in total β-catenin expression (measured as the ratio of total β-catenin per AER divided by total perimeter) (0.44±0.06 vs. 1.01±0.09, p = 0.005, Fig. 9I). This global decrease was associated with specific decrease in β-catenin expression at the membrane (measured as the ratio of β-catenin in the perimeter over total perimeter) (14%±0% vs. 49%±4%, p = 0.005 Fig. 9J) but unchanged total β-catenin in the cytoplasm (measured as the ratio of cytoplasmic β-catenin over total β-catenin) (0.46±0.04 vs. 0.53±0.01, p = 0.20; Fig. 9K). Altogether, these data are in harmony with impaired β-catenin signaling following massive β-catenin destabilization. Quantification of these parameters at 6 hrs (n = 3) indicated a restoration of the total level of β-catenin compared to 0 hr (Fig. 9I). However, the presence of β-catenin in the plasma membrane pool is still reduced (30%±2% vs. 49%±4%, p = 0.005 Fig. 9J) while the percentile of cytoplasmic pool is unchanged (Fig. 9K) suggesting that the adhesive properties of the AER cells are still perturbed at 6 hrs Dox-IP.

## Discussion

### FGFs play important roles in vertebrate limb development

*Fgf10* is the main *Fgfr2b ligand* expressed in the limb between E10.5 and E12.5 (Fig. S5) suggesting that our transgenic system allows addressing specifically the early role of FGF10 signaling to the Apical Ectodermal Ridge via FGFR2b. Indeed, FGFR2b acts as the main receptor for FGF10 during limb development as evidenced by the absence of limbs in both *Fgf10* and *Fgfr2b* null embryos [5], [21], [22]. Previous studies using [*Msx2-cre;Fgfr2^fl/fl^*] mouse to conditionally delete *Fgfr2* in the AER reported the loss of autopod in the forelimbs, and agenesis of hindlimbs [8]–[9]. However, these studies failed to delineate the role of FGFR2b signaling at different stages during the pre and post-limb bud induction, since the authors did not use an inducible Cre system. Here we show that these are rendered possible by the use of an inducible and reversible mouse model in which signaling induced by FGFR2b-ligands interaction is inhibited after exposure to Doxycycline. In this in vivo model, a transactivator rtTA is expressed constitutively from the ubiquitous *Rosa26 (R26)* locus, leading to the ubiquitous expression of soluble dominant-negative FGFR2b molecules. These act by sequestering all FGFR2b-activating ligands, including FGF10, away from cell surface-expressed FGFR2b. Indeed, soluble FGFR2b molecules are as effective as genetic ablation of *Fgfr2b* and *Fgf10* [3]–[5], [10].

### Attenuation of FGFR2b-ligands interaction using the DTG system is fully reversible

**Figure 10:**
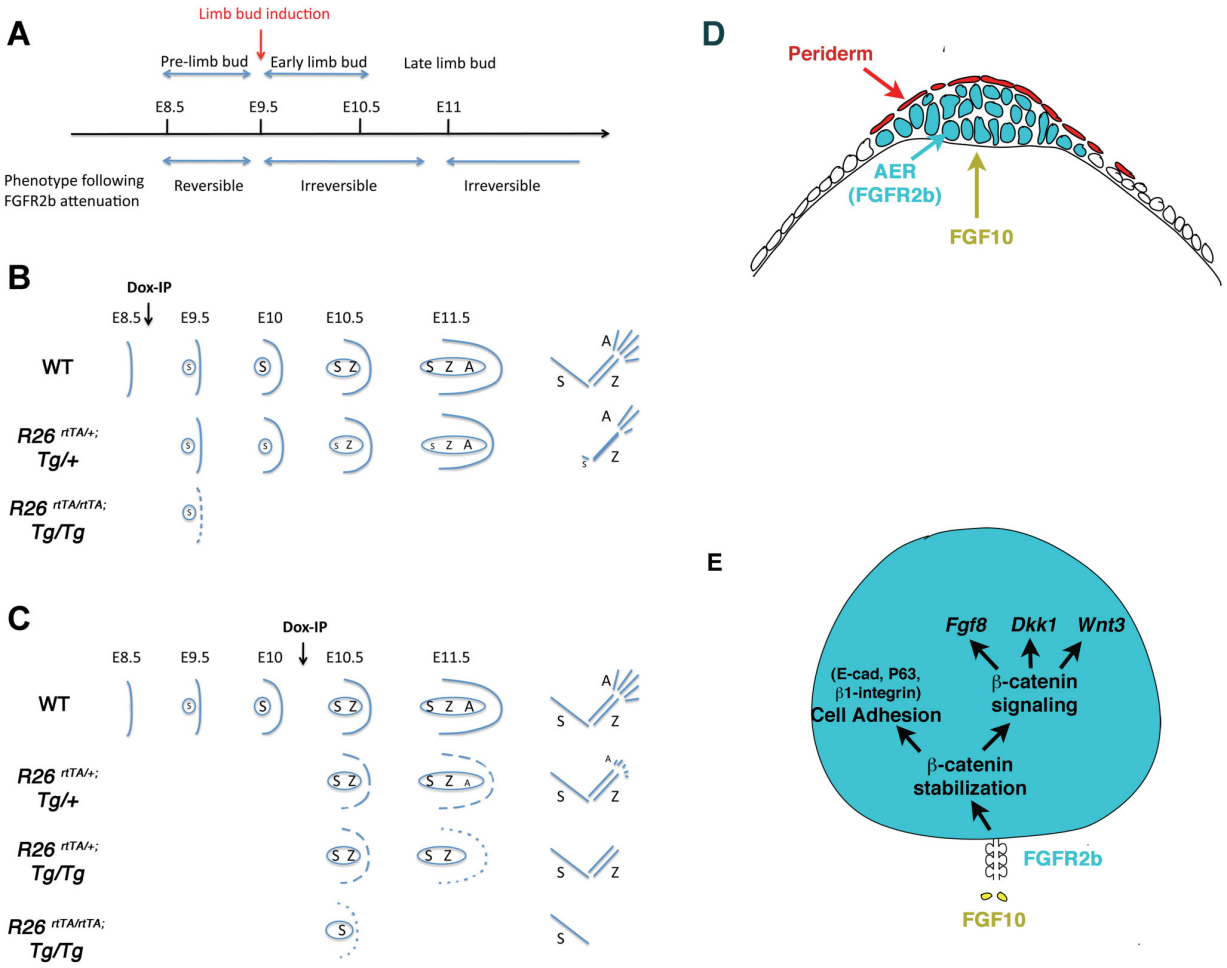
Signaling induced by FGFR2b-ligands interactionplays a critical function to control the amplification of the mesenchymal progenitors throughout limb development. (**A**) FGFR2b-ligands signaling plays a major function in the amplification of the mesenchymal cells that will give rise to the stylopod prior to limb bud induction. Transient inhibition of FGFR2b-ligands signaling at E8.5 is compatible with limb induction while inhibition at E9.5, 10.5 and E11.5 leads to the irreversible loss of the AER. (**B**) Consequences of Dox-IP at E8.5. In double heterozygous embryos, the mesenchymal progenitors that are specified to give rise to the stylopod are not amplified but the AER is induced at E9.5 allowing the formation and amplification of the mesenchymal progenitors for the *zeugopod* and autopod. Note that in our model the efficiency of the AER may not be the same as in WT as progenitors for Z and A are not amplified at the same rate leading to corresponding skeletal defects. In double homozygous embryos, the stylopod progenitors are not amplified and the AER is not functional (dotted line) leading to limb agenesis. (**C**) Consequences of Dox-IP at E10/E10.5. In double heterozygous embryos, the AER is lightly affected at E10.5 (dotted lined with big distances between gaps) the progenitors for the autopod are partially amplified leading to shorter digits. In *[R26^rtTA/+^; Tg/Tg]* embryos, the AER is mildly affected at E10.5 (dotted line with smaller distance between gaps) the progenitors for the autopod are not amplified at all leading to absence of autopod. In double homozygous embryos, the AER is no longer functional at E10.5 (dotted line) and leads to absence of amplification of the mesenchymal progenitors for the zeugopod and autopod). (**C**) Schematic representation of the AER showing the superficial layer of periderm cells covering the compact AER cells expressing FGFR2b. FGFR2b ligand, FGF10 is expressed in the underlying mesenchyme. (**D**) FGFR2b signaling in the AER cells allows β-catenin stabilization. Such stabilization downstream of FGFR2b signaling, allows β-catenin function in cell adhesion and in signaling, *Fgf8*, *Dkk1* and *Wnt3* are known downstream targets.

During previous studies of postnatal mammary gland and adult tooth, we found that our DTG system to attenuate FGFR2b-ligands signaling is not leaky and fully reversible [10], [23]. The present results also support reversibility in soluble *Fgfr2b* expression and activity during embryonic development. First, Dox-IP administration to *R26^rtTA/rtTA^;Tg/Tg* embryos at E11 leads to a peak in *soluble Fgfr2b* expression 6 hours later with a return to almost base line level at 24 hours. When the injection occurs at E8.5, this translates into inhibition of the AER in the forelimb (which is formed at E9.5, 24 hrs after the Dox-IP injection) and partially disrupted AER in the hindlimb (which is formed at E10, 36 hrs after the Dox-IP injection). We therefore conclude that the activity of the soluble FGFR2b protein in this particular allelic combination, which allows for the highest level of soluble FGFR2b expression, lasts around 36 hours. Second, Dox-IP administration to [*R26^rtTA/rtTA^;Tg/Tg*] embryos at E7.5 leads to completely normal limb formation at E18.5 (data not shown), which is consistent with the 36 hours inhibition period ending at E9.0, 12 hrs and 24 hrs before the induction of the AER in the forelimb and hindlimb, respectively. Third, Dox-IP administration to [*R26^rtTA/+^;Tg/+*] embryos at E8.5, an allelic combination which allows for the lowest level of soluble FGFR2b, leads to relatively mild forelimb defects (missing stylopod) and a nearly normal hindlimb (Fig. 10B) suggesting that the activity of soluble FGFR2b is minimal at 24 hours post Dox injection (at the time of forelimb AER induction) and absent at 36 hours post Dox-IP (at the time of hindlimb induction). We can therefore conclude that the expression of soluble *Fgfr2b* in our transgenic system is not leaky and is inducible and reversible. In addition, the different allelic combinations allow for a temporal inhibition of FGFR2b signaling up to 24 hours for the lower doses of *sFgfr2b* or up to 36 hours for the higher doses of *sFgfr2b* following a single Dox-IP injection. Interestingly, the comparison of the different phenotypes in heterozygous versus homozygous between transient (Fig. 2) and sustained (Fig. 1) FGFR2b-ligands attenuation suggests that as the dose of soluble FGFR2b increases, more proximal truncations are generated (Fig. 10B). This is the likely consequence of defective mesenchymal expansion occurring at the time of soluble FGFR2b induction. It seems therefore that soluble FGFR2b dosage is translated into a temporal factor that corresponds to the amplitude and duration of FGFR2b inhibition (Fig. 10C).

### Inducible FGFR2b-ligands signaling attenuation allows the characterization of the early changes impacting the AER at the cellular and molecular levels

The rapid disappearance of the AER in response to FGFR2b-ligands signaling attenuation via the DTG system allows for fine dissection of the mechanisms by which interaction of FGFR2b with its ligands impacts the AER and its knock-on effects on adjacent mesenchymal cell population. The timing of FGFR2b-ligands inactivation in the DTG system is fundamentally different from the one aiming to conditionally delete *Fgfr2* expression using the *Cre/Lox* technology [8] [9]. In the latter, the function of existing FGFR2 molecules is unperturbed at the onset of genetic ablation. Thus, for example, it takes at least 24 hours, from the onset of Cre expression in the AER using the *Msx2-Cre* driver line, to see the complete disappearance of the AER (from stage 29 through 45 roughly corresponding to E10.5 through E11.5 [8]). The presented phenotypes therefore depend not only on the timing of Cre expression but also on the stability and turnover of the pre-existing FGFR2b molecules.

Previous reports have described the mechanism of AER formation; In particular Altabef et al [24]–[25] have shown that AER is formed by the convergence of ectodermal cells at the presumptive dorso-ventral boundary of developing limb buds. This convergence suggests that the AER-fated ectodermal cells acquire adhesive characteristics that distinguish them from their neighbors to eventually form a compact multistratified epithelium. The formation of protein complexes containing cytoskeletal proteins, receptor tyrosine kinase and extracellular ligands is often organized around Integrins and Cadherins [26]. Our study is the first to analyze the process of AER maintenance downstream of FGFR2b-ligands signaling. We found that β1-integrin, E-cadherin and P63 expression were reduced upon blockade of FGFR2b-ligands and this was accompanied by a relatively rapid and dramatic remodeling of the multilayered AER epithelium (Fig. 10E). It remains to be determined whether the AER cells lose their identity and become fully incorporated into the overlying ectoderm, or retain some of their original characteristics. Interestingly, embryos that are deficient in both α3 and α6- integrins exhibit a flattened ridge containing cells that lack the usual columnar morphology. In addition, epithelial cell proliferation was reduced in the AER and alterations of the basal lamina underlying the ectoderm were observed [27]. Overall, these results suggest that integrins are required for the organization and/or compaction of the ectodermal cells in the AER. Our results indicate that FGFR2b-ligands signaling to the AER potentially works upstream of integrins to control the formation and maintenance of such a distinctly differentiated structure. Rapid changes in cell morphology are usually caused by remodeling of cell cytoskeleton (e.g. microtubule reassembly) and so FGFR2b-ligands signaling could also lay directly upstream of these mechanisms.

In terms of appearance and structure, AER cells appear to recover 2 hours after a single Dox-IP dose at E11.0. However, data in Figure 2, 3 and S2 indicate that this apparently “recovered” AER is not functional. The cellular and molecular processes controlling the initial recovery of the AER (2–4 hrs time point) during development are unknown and will require further investigation.

### FGF and WNT signaling control de novo AER induction

Our data indicate that restoration of FGFR2b-ligands signaling alone is not sufficient to induce de novo AER signaling. Using the *Topgal* reporter as a read out for WNT signaling at different times after Dox-IP, we show that WNT-AER signaling is still impaired at the 24 hour time point, where soluble FGFR2b activity is negligible or absent. We propose that this is likely the underlying cause of lack of de novo AER induction. Interestingly, using the *Msx2-Cre* driver line, Lu et al [8] reported that the impact of *Fgfr2* deletion on the maintenance of the AER could be rescued by the concomitant expression of a stable form of β-catenin. Even though this rescue experiment does not allow us to conclude that WNT signaling activation after a transient inhibitory period (24 hour) is sufficient to induce de novo AER formation, it is likely that the stabilization of β-catenin at significant levels is the limiting factor for de novo AER formation (Fig. 10E). Furthermore it indicates that FGFR2b-ligands signaling to the AER, even though it is *per se* capable of directly activating β-catenin signaling, is not enough to provide sufficient activation of this pathway to re-engage the cellular and molecular mechanisms in the ectodermal cells to reform the specialized AER structure. In support of this conclusion, it has recently been shown that AER can be ‘re-induced’ by insertion of WNT2b and FGF10-expressing cells into wounded chick limbs [28]. Interestingly, the AER can be re-induced only in wounded limbs but not in intact limbs. The molecular basis for this difference is unclear. Thus, FGF signaling in combination with WNT signaling are likely to be instructive in this process of de novo AER formation. However, our data also indicates a strict requirement for FGFR2b-ligands signaling in the maintenance of the AER throughout the bud stage of limb development (Fig. 2–3).

### A series of WNT ligands are involved in WNT-AER signaling

Conclusions regarding the respective importance of FGF versus β-catenin signaling in the AER can be drawn based on the limb phenotype of [*Msx2-Cre; Fgfr2^flox/flox^*] versus [Msx2-Cre; *β-cat^flox/flox^*] mutant embryos, with the latter being more severe than the former. While both mutants exhibit hindlimb agenesis, the forelimbs are differently affected. [*Msx2-Cre; Fgfr2^flox/flox^*] exhibit autopod agenesis while [*Msx2-Cre; β-cat^flox/flox^*] mutant forelimbs fail to form the zygopod (ulna radius) and the autopod. Our results indicate that FGF10/FGFR2b signaling can directly activate β-catenin signaling in HEK293T cell line. We believe that even if this is the case during limb development, other growth factors, such as WNT ligands, are also contributing to WNT-AER signaling. *Wnt3* in mouse has been identified as a critical gene for AER maintenance during limb development [20]. The limb phenotype of [*Msx2-Cre; Wnt3^flox/flox^*] animals ranged from completely normal to completely absent hindlimb, with most of the hindlimbs exhibiting mild to severe autopod defects or more extensive truncations that extended into more proximal segments of the limb. Interestingly, these mutant embryos exhibited seemingly normal forelimb, suggesting that other WNT ligands are at work to compensate for the lack of *Wnt3* in the forelimb or that FGF10/FGFR2b signaling in the forelimb is enough to compensate the loss of *Wnt3* expression. Suggesting slightly different activities for FGFR2b signaling in the hindlimb vs. forelimb during development, we found that *Fgf8* expression is still maintained in the forelimb 24 hrs Dox-IP (Fig. 4H) while it is completely absent in the hindlimb at this stage. This conclusion appears to also apply for *Wnt3*. However, our *Wnt* qRT-PCR array showed that many proximal mesenchymal *Wnt* target genes are affected when FGFR2b-ligands signaling is hindered. The changes in these different genes disrupts β-catenin signaling, thus preventing the maintenance of the AER.

### FGFR2b-ligands signaling is not sufficient to induce de novo AER formation after transient FGFR2b-ligands inactivation

Figure 8P indicated that the endogenous *Fgf10* expression level is not changed upon FGFR2b-ligands inactivation and Figure 3H shows that the expression of endogenous *Fgfr2b* is increased at 24 hours following Dox-IP administration. In addition, the analysis of the E18.5 limb phenotype following a single IP injection in pregnant females carrying E8.5 *[R26^rtTA/+^; Tg/+]* embryos suggest that the expression/activity of soluble *Fgfr2b* is very low at the 24 hours time point (which corresponds to the induction of the AER in the forelimb at E9.5) and negligible at the 36 hours time point (which corresponds to the induction of the AER in the hindlimb at E10). Altogether these results are pointing out to a scenario where both FGF10 and FGFR2b are expressed normally 24 hours after Dox-IP with a residual presence of soluble FGFR2b at 24 hrs and complete absence of the inhibitor at 36 hrs. We conclude that the activation of the endogenous FGFR2b/FGF10 pathway is restored 24 hrs after Dox IP but that this is not sufficient for de novo AER formation. Interestingly, our results indicated that the WNT pathway is still inhibited after 24 hrs (Fig. S3) suggesting that one or several critical elements of the WNT pathway is/are missing at this stage, in spite of the restoration of the endogenous FGF10/FGFR2b pathway. In addition to the *Wnt* ligands described in the previous section, our results point out to irreversible cellular localization defects in β-catenin per se in the AER cells correlating with the continuous lack of β-catenin signaling observed in our studies. This is likely the main reason underlying the lack of de novo AER formation. The fate of these β-catenin-impaired AER cells is still unclear. It is possible that these cells are now incorporated as part of the squamous epithelium. At this point, restoration of endogenous FGF10/FGFR2b signaling during embryonic limb development is not sufficient to change the fate of these squamous epithelial cells back to the AER fate.

In conclusion, our findings show that FGFR2b-ligands signaling has critical stage-specific roles in maintaining the AER during limb development

## Materials and Methods

### Ethics Statement

Animal experiments were performed under the research protocols (31-08 and 31-11) approved by the Animal Research Committee at Children's Hospital Los Angeles and in strict accordance with the recommendations in the Guide for the Care and Use of Laboratory Animals of the National Institutes of Health. The approval identification for Children's Hospital Los Angeles is AAALAC A3276-01.

### Animals

To generate the inducible mouse model, which expresses soluble FGFR2b, *CMV-Cre* mice were first crossed with *rtTA^flox^* mice [29]. The resulting *Rosa26^rtTA/+^* (*R26^rtTA/+^*) mice were crossed with *tet(O)sFgfr2b* (*Tg*) mice [30] for several generations on a mixed background. Different allelic combinations for the *R26^rtTA^* and the *tet(O)Fgfr2b (Tg)* transgene ([*R26^rtTA/+^;Tg/+^−^*], [*R26^rtTA/+^;Tg/Tg*] and [*R26^rtTA/rtTA^;Tg/Tg*]) were generated to allow the expression of different levels of soluble FGFR2b following doxycycline (Dox) treatment. These mice are called DTG for simplification but the exact genotype is specified. The control embryos are the wild type and single transgenic littermates of the DTG embryos. The *Topgal* mouse model was previously described by DasGupta and Fuchs [11]. Animals were crossed and time pregnant females were either put on Dox food (Rodent diet with 0.0625% Dox, Harlan Teklad TD01306, Hayward, CA, USA) for several days or injected intraperitoneally (IP) with a single dose of Dox (1.5 mg/kg of mouse in PBS) at a specific developmental stage. Pregnant females were euthanized to collect embryos at different stages. All samples were fixed in 4% Paraformaldehyde (PFA) and dehydrated in successive batches of increasing ethanol concentration for further studies.

### β-galactosidase staining

β-galactosidase/LacZ staining was performed as previously described [32], [31]. Briefly, control-*Topgal* and DTG-*Topgal* embryos were fixed one hour in 4% PFA. Following the fixation, the embryos were washed in PBS and incubated overnight at 37°C with the LacZ buffer solution containing 40 mg/mL of X-gal (rpi research products, Mount Prospect, IL, USA).

### Bone and Cartilage Staining

Staining of the skeletons was carried out using minor modifications of a previously described protocol [32]. Embryos were collected at various stages, skinned and eviscerated. The samples were fixed in 95% ethanol for three days, changing ethanol every day and subsequently, stained for cartilage in 0.3% alcian blue 8GS in freshly prepared staining solution at room temperature for two days. Embryos were then washed in distilled water for two hours and stained in 0.2% alizarin red S in 0.5% KOH for several hours at room temperature. Samples were then washed for several days in 0.5% KOH to remove excess color and transferred to 0.5% KOH/20% glycerol and photographed under a Stemi2000 Zeiss microscope. For long-term storage, samples were transferred to 80% glycerol 0.5% KOH.

### Immunofluorescence

Limb buds were embedded in paraffin sectioned and stained for various analyses. Apoptotic cells were detected by incorporation of terminal deoxynucleotidyltransferase mediated UTP nick-end labeling (TUNEL) using the “In Situ Cell Death Detection, Fluorescein kit (Roche Applied Science, Indianapolis, IN, USA) as recommended by the manufacturer or by immunofluorescent staining with cleaved caspase 3 antibody (1∶100, cell signaling, Danvers, MA, USA). The total number of cells was scored in five photomicrographs (40× magnification) of four independent control and mutant limbs. A total number of 100 cells were counted per sample. For immunofluorescence staining, paraffin sections were stained with primary antibodies against mouse P63 (1∶200, D9, Santa Cruz Biotechnologies, Santa Cruz, CA, USA) and β1 integrin (1∶100, Millipore, Billerica, MA, USA), E-cadherin (1∶100, BD Biosciences, Franklin Lakes, NJ, USA), Phospho-histone H3 (1∶200, Cell signaling, Danvers, MA, USA) and β-catenin (1∶200, BD Biosciences, San Jose, CA, USA). FITC- and Cy3- conjugated secondary antibodies (1∶200, Jackson Immunoresearch Laboratories, Inc., West Grove, PA, USA) were used; the slides were then mounted with Vectashield containing DAPI. Fluorescent images were acquired using a Leica monochrome camera attached to a Leica DM4000B microscope.

### Quantification of fluorescence signal for β-catenin

The AER and cell boundaries were delineated manually with single-pixel lines using the pencil tool in Photoshop (Adobe Systems Inc., San Jose, CA). Then, internal and peripheral β-catenin signal were quantified using Fiji ImageJ [33] by first expanding the lines with 2 iterations of a morphological dilate operation and then measuring integrated intensity of the red channel that coincides with the expanded lines. Total integrated red intensity within the outer boundaries of the AER was also measured. To quantify the percent distance of cell boundaries covered by β-catenin, all boundary pixels and the pixels containing β-catenin staining above a threshold of 44 were summed separately.

### Scanning Electron Microscopy

Mouse embryos were extracted quickly from the uteri, washed 6 times in filtered PBS and fixed in a solution of sodium cacodylate 0.1 M pH 7.6/glutaraldehyde 2% at room temperature for 1 hour and then overnight at 4°C. They were washed three times in 0.2 M sodium cacodylate for 1 hour at room temperature and transferred in a solution of sodium cacodylate 0.1 M pH7.6/OsO4 0.1% for 1 hour at room temperature. After a 5-minute wash in distilled water, the embryos were dehydrated in graded ethanols (70% to 100%) and then in amyl acetate (30% to 100%). They were critical point dried in liquid carbon dioxyde, mounted on aluminum stubs and coated with gold.

### Transmission Electron Microscopy

The embryos were fixed with 2% glutaraldehyde in 0.1M phosphate buffer, post-fixed with 1% OsO4 in 0.1M phosphate buffer, dehydrated with ethanol, and embedded in Epon with careful orientation. Semi-thin sections were cut and stained with staining solution (azure II, methylene blue, sodium borate, and basic fuscin), and reviewed with a light microscope for selecting the areas of interest for ultrastructural examination. Ultra-thin sections were cut onto the grids and stained with uranyl acetate and a mixture of lead nitrate, lead acetate, and lead citrate. Ultrastructural images were examined with a transmission electron microscope (Morgagni 268), and selected areas were digitally recorded.

### Whole Mount In Situ Hybridization

Embryos were fixed in 4% PFA, washed and dehydrated. In situ hybridization was performed with digoxigenin labeled UTP RNA *Fgf8* (a gift from Dr. F. Mariani), *Wnt3* (a gift from Dr. A. McMahon), *Dkk1* (a gift from Dr. C. Niehrs), *Meis 1, Hoxa11, Hoxa13* (gift from Dr. X. Sun) AS probes according to a modified protocol from Bellusci et al. [34]. Sense probes were used to verify specific hybridization of the AS probes.

### Real time PCR

RNA was extracted from either hindlimbs or forelimbs of transgenic animals injected with Doxycycline using the iNtRON Biotechnology, Inc. easy-spin^TM^ Total RNA Extraction Kit. RNA (500 ng/µl) was reverse-transcribed into cDNA using Transcriptor First Strand cDNA Synthesis Kit (Roche Applied Science) according to the manufacturer's instructions. cDNA (1 µg) was used for dual color Hydrolysis Probe-Universal probe library based real time PCR, using the LightCycler 480 from Roche Applied Science. *Gapdh* assay commercially available from Roche Applied Science was used as reference gene. See the supplemental data section for the details on the primers and Roche Applied Science Universal probes used for each of the assayed genes (Table S1).

### Statistical Analysis

Statistical analyses were performed using Statview software version 4.57.0.0. All data are expressed as means ± SEM. For each of the experiments, at least n = 4 of each condition was used. Comparisons of the changes between controls and DTG were performed using the nonparametric Mann-Whitney test. A p<0.05 was considered significant.

## Supporting Information

Figure S1 **Digit defects after inhibition of FGFR2b-ligands signaling from E11.5 to E18.5** leads to digit defects in the forelimb (A,C,E) and hindlimb (B,D,F) of both [*R26^rtTA/+^; Tg/Tg*] (C,D) and [*R26^rtTA/rtTA^; Tg/Tg*] (E,F) embryos compared to wild types limbs (A,B).
(TIF)

Figure S2 **Vascular development is not impaired upon FGFR2b-ligands attenuation.** (**A–D**) expression of PECAM by IF in control (A,B) and (C,D) 6 hrs Dox-IP limbs. (**E, F**) Quantification of *Pecam* and *Vegfa* expression by qRT-PCR. Scale bars: A–D: 50 µm.
(TIF)

Figure S3 **Inhibition of WNT signaling in vivo upon FGFR2b-ligands attenuation.** E11 [*R26^rtTA/+^; Tg/+; Topgal]* embryos are analyzed at 0 (A–C), 2 (D–F), 12 (G–I) and 24 (J–L) hrs after Dox-IP. Staining for *Topgal* shows a massive inhibition of WNT signaling persisting at least up to 24 hrs after Dox-IP. (M–O) E12 [*R26^+/+^; Tg/+; Topgal]* embryo showing *Topgal*/WNT signaling activation in the whiskers, AER and mammary buds. Scale bars: A–O: 500 µm.
(TIF)

Figure S4 **FGF10 directly activates the β-catenin pathway.** HEK293T cells were transfected with pRLTK (Renilla) together with TOPFLASH and subsequently incubated in DMEM containing low serum (0.2% FCS). Cells were treated with either media alone, FGF1 (10 ng/ml), FGF10 (250 ng/ml), Wnt3A (10 ng/ml) or LiCl (20 mM) for 1 and 6 hours. Cells were lysed and assayed for luciferase activity. Values are depicted as means ± SEs of cells treated with media alone and are from three experiments.
(TIF)

Figure S5 ***Fgf10*** **is the main** ***Fgfr2b ligands*** **expressed during limb bud development.** The expression of *Fgf1*, *3*, *7* and *10* was investigated by qPCR at E110.5, E11.5 and E12.5 in dissected forelimbs (n = 3).
(TIFF)

Table S1 List of primers and probes for qRT-PCR.
(DOCX)

Table S2 Genes up and down regulated in the WNT pathway.
(DOCX)
