# Supplementary figures and images for: Transient Inhibition of FGFR2b-Ligands Signaling Leads to Irreversible Loss of Cellular β-Catenin Organization and Signaling in AER during Mouse Limb Development
Source: PLoS One. 2013 Oct 22;8(10):e76248. doi: 10.1371/journal.pone.0076248 (PMC3805551; doi:10.1371/journal.pone.0076248)

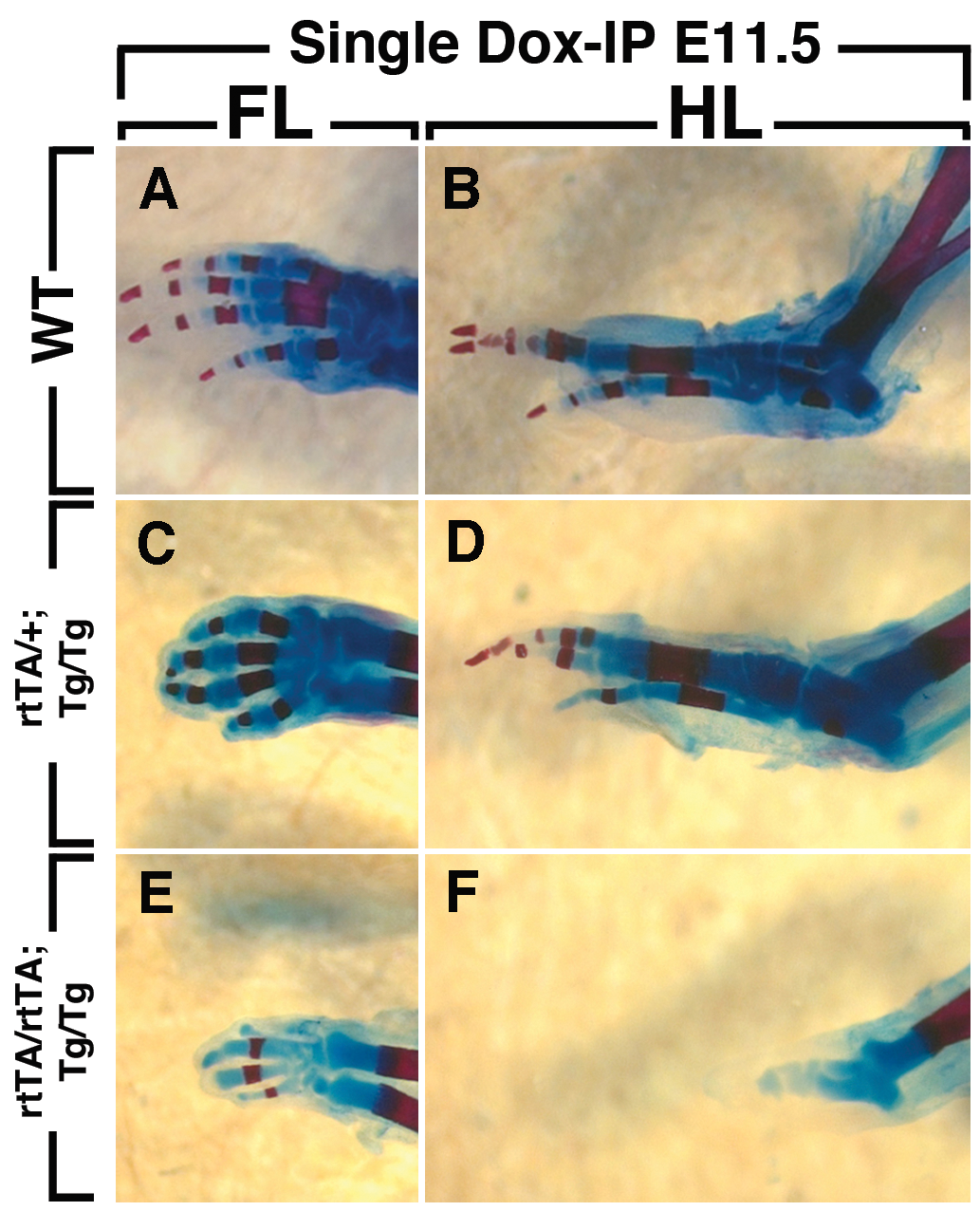

Supplement: Figure S1 — Digit defects after inhibition of FGFR2b-ligands signaling from E11.5 to E18.5 leads to digit defects in the forelimb (A,C,E) and hindlimb (B,D,F) of both [R26rtTA/+; Tg/Tg] (C,D) and [R26rtTA/rtTA; Tg/Tg] (E,F) embryos compared to wild types limbs (A,B). (TIF) [file pone.0076248.s001.tif]

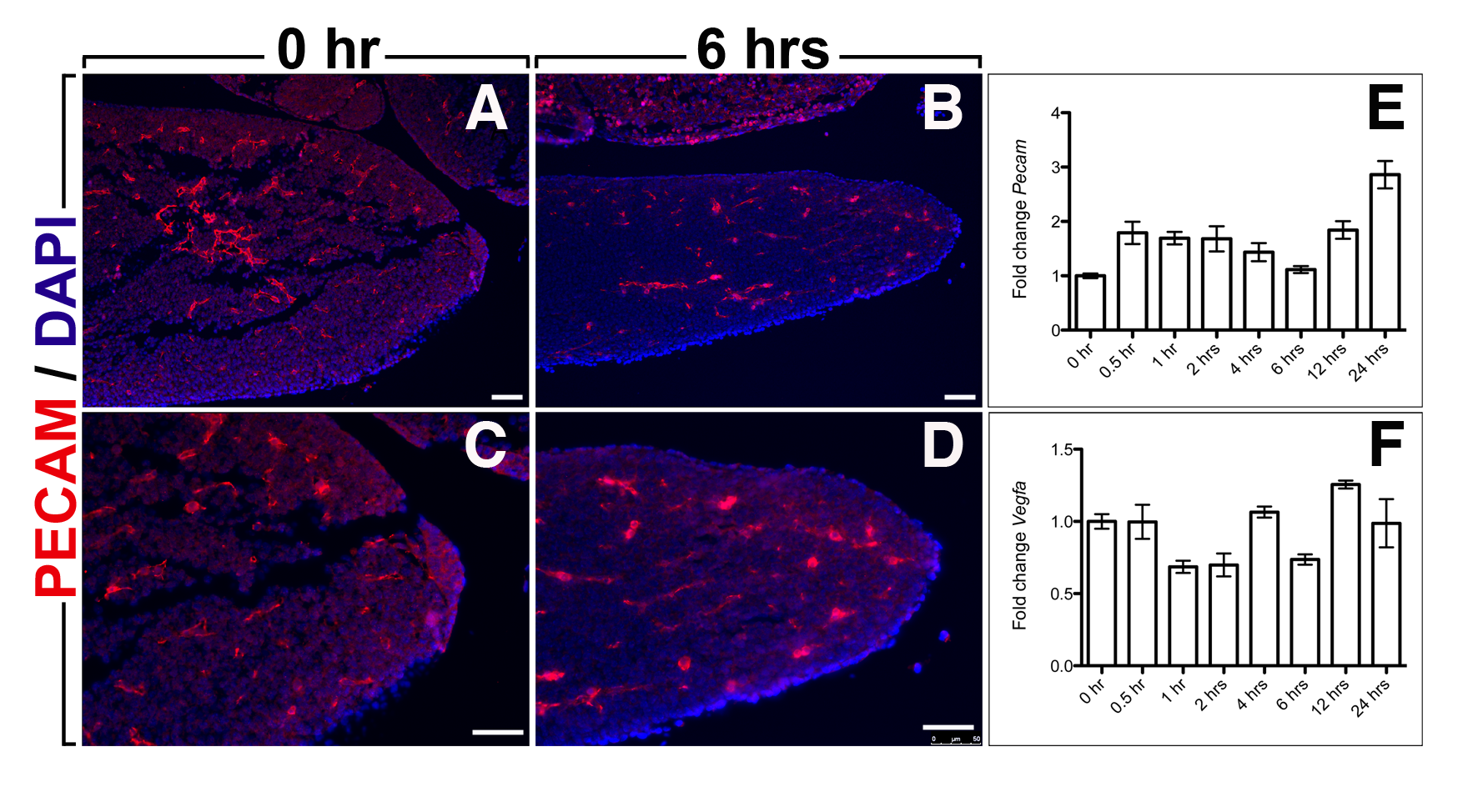

Supplement: Figure S2 — Vascular development is not impaired upon FGFR2b-ligands attenuation. (A–D) expression of PECAM by IF in control (A,B) and (C,D) 6 hrs Dox-IP limbs. (E, F) Quantification of Pecam and Vegfa expression by qRT-PCR. Scale bars: A–D: 50 µm. (TIF) [file pone.0076248.s002.tif]

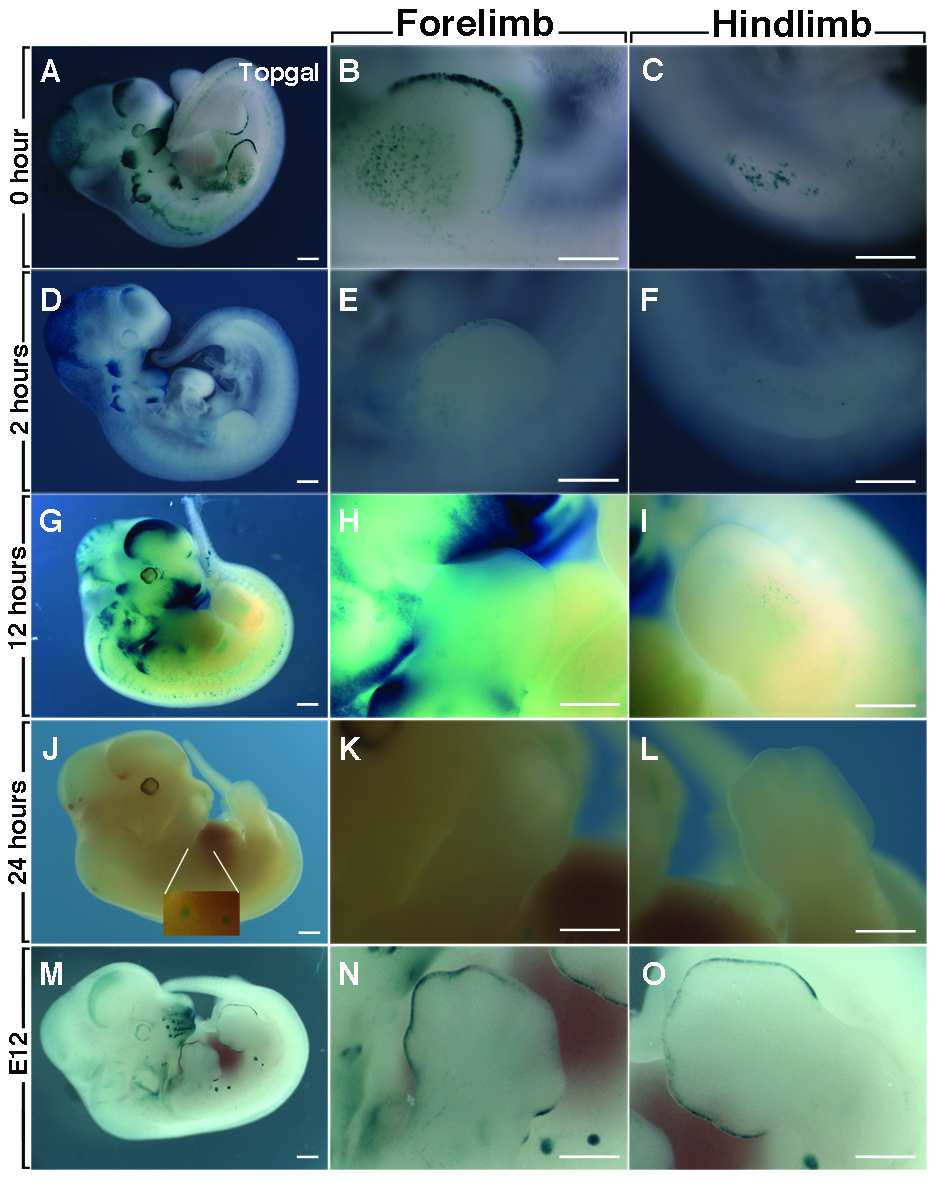

Supplement: Figure S3 — Inhibition of WNT signaling in vivo upon FGFR2b-ligands attenuation. E11 [R26rtTA/+; Tg/+; Topgal] embryos are analyzed at 0 (A–C), 2 (D–F), 12 (G–I) and 24 (J–L) hrs after Dox-IP. Staining for Topgal shows a massive inhibition of WNT signaling persisting at least up to 24 hrs after Dox-IP. (M–O) E12 [R26+/+; Tg/+; Topgal] embryo showing Topgal/WNT signaling activation in the whiskers, AER and mammary buds. Scale bars: A–O: 500 µm. (TIF) [file pone.0076248.s003.tif]

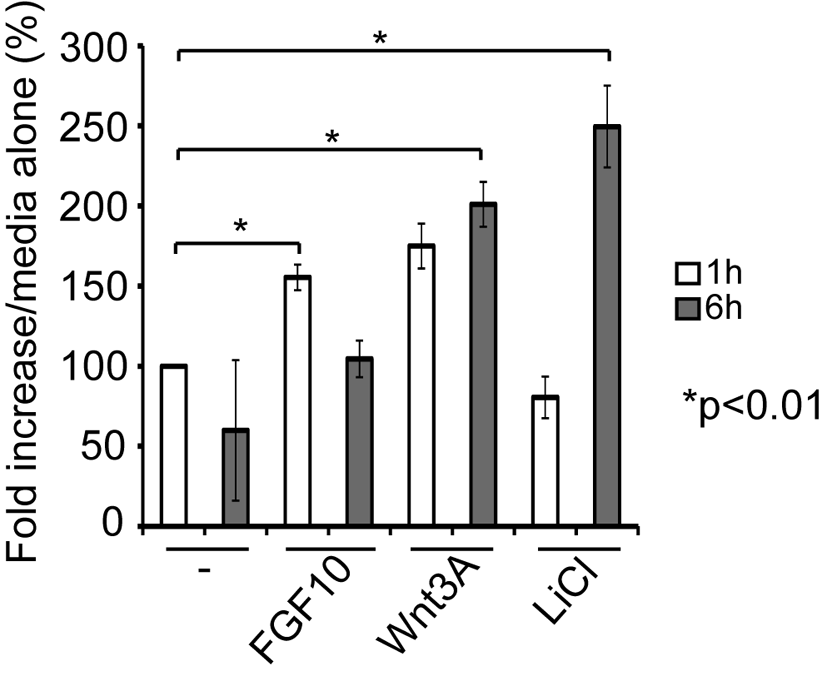

Supplement: Figure S4 — FGF10 directly activates the β-catenin pathway. HEK293T cells were transfected with pRLTK (Renilla) together with TOPFLASH and subsequently incubated in DMEM containing low serum (0.2% FCS). Cells were treated with either media alone, FGF1 (10 ng/ml), FGF10 (250 ng/ml), Wnt3A (10 ng/ml) or LiCl (20 mM) for 1 and 6 hours. Cells were lysed and assayed for luciferase activity. Values are depicted as means ± SEs of cells treated with media alone and are from three experiments. (TIF) [file pone.0076248.s004.tif]

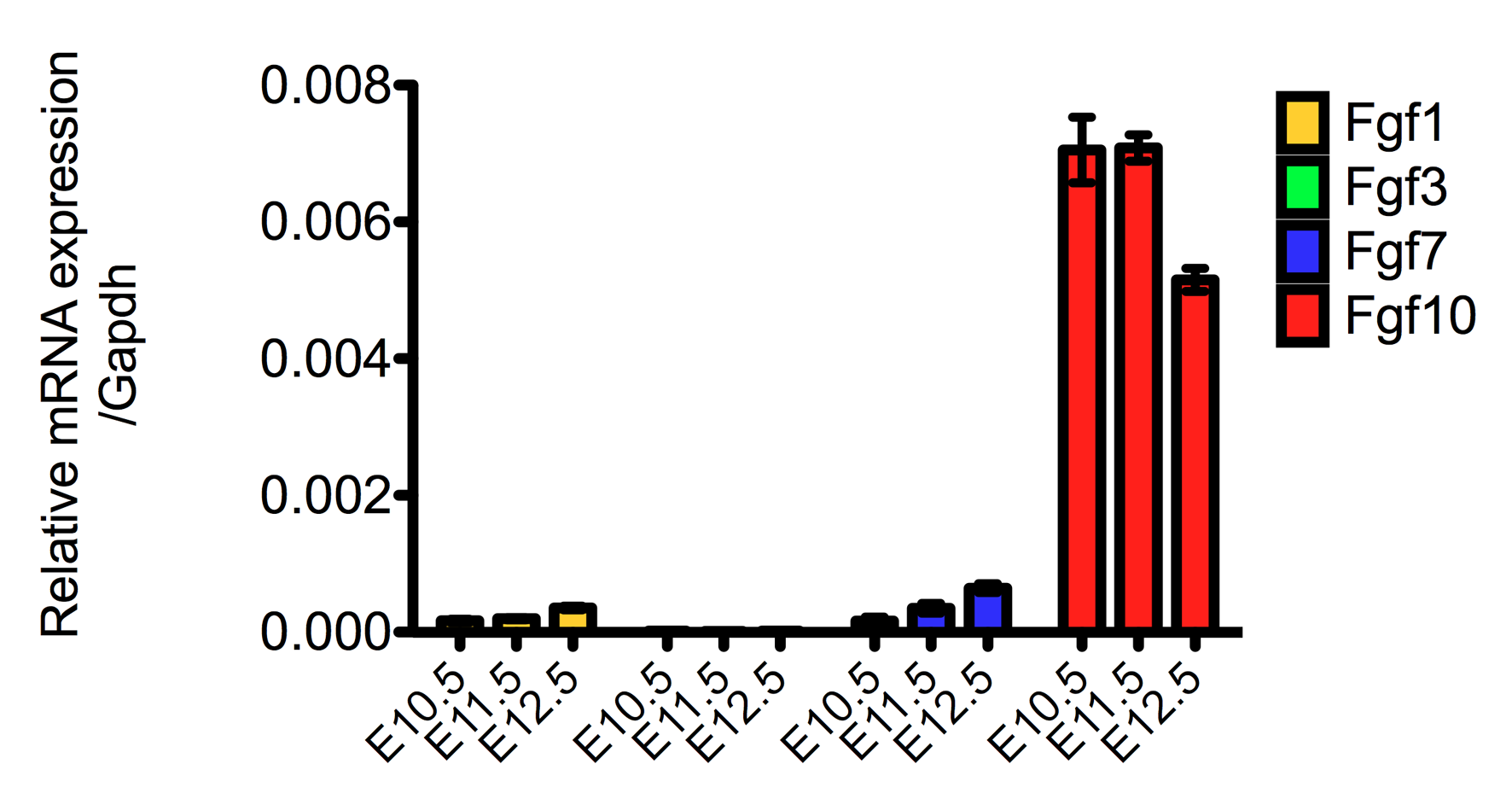

Supplement: Figure S5 — Fgf10 is the main Fgfr2b ligands expressed during limb bud development. The expression of Fgf1, 3, 7 and 10 was investigated by qPCR at E110.5, E11.5 and E12.5 in dissected forelimbs (n = 3). (TIFF) [file pone.0076248.s005.tif]
