# Supplementary material for: Transient Inhibition of FGFR2b-Ligands Signaling Leads to Irreversible Loss of Cellular β-Catenin Organization and Signaling in AER during Mouse Limb Development
Source: PLoS One. 2013 Oct 22;8(10):e76248. doi: 10.1371/journal.pone.0076248 (PMC3805551; doi:10.1371/journal.pone.0076248)
Supplement: Table S1 — List of primers and probes for qRT-PCR. (DOCX) [file pone.0076248.s006.docx]

**Table S1 qRT-PCR**

Primers and Roche Applied Science Universal probes used for each of the assayed genes:

| Gene | Probe | Primer Orientation | Sequence |
| --- | --- | --- | --- |
| *Axin2* | 50 | Right | TGCCAGTTTCTTTGGCTCTT |
|  |  | Left | CTGCTGGTCAGGCAGGAG |
| *Beta-1-Integrin* | 18 | Right | GTCCCACAGTACAGCCCTTG |
|  |  | Left | GTGCCAATCTTTCATGATGC |
| *Dkk1* | 76 | Right | CCAAGGTTTTCAATGATGCTT |
|  |  | Left | CCGGGAACTACTGCAAAAAT |
| *E-Cadherin* | 18 | Right | ACCACCGTTCTCCTCCGTA |
|  |  | Left | ATCCTCGCCCTGCTGATT |
| *Fgf8* | 32 | Right | TGAGCTGATCCGTCACCA |
|  |  | Left | TCCTGCCTAAAGTCACACAGC |
| *Fgf10* | 80 | Right | AACAACTCCGATTTCCACTGA |
|  |  | Left | CGGGACCAAGAATGAAGACT |
| *Endogenous Fgfr2b* | 21 | Right | CATCCATCTCCGTCACATTG |
|  |  | Left | CCCTACCTCAAGGTCCTGAA |
| *Soluble Fgfr2b* | 108 | Right | GAAGGAGATCACGGCTTCC |
|  |  | Left | AGACAGATGATACTTCTGGGACTGT |
| *Frzb* | 34 | Right | CACCGTCAATCTTTATACCACCT |
|  |  | Left | TCAGCTATAGAGCCTTCTACCAAGA |
| *Fzd4* | 20 | Right | AACTTTCACGCCGCTCAT |
|  |  | Left | CCGAACAAAGGAAGAACTGC |
| *Fzd8* | 105 | Right | TGCTCAAGTACTTCATGTGCCTA |
|  |  | Left | CAGAGTCTTGCCGGACCA |
| *Fzd9* | 4 | Right | TTTCTTCTCCACGGCCTTC |
|  |  | Left | GGTACTGGAACCGGTGAGG |
| *Hoxa11* | 72 | Right | CCGCAGGCATTTCCTGTA |
|  |  | Left | GAAAACTCCAGAGGCTGTGC |
| *Hoxa13* | 75 | Right | TCTGAAGGATGGGAGACGAC |
|  |  | Left | TACTGCCCCAAAGAGCAGA |
| *Meis1* | 103 | Right | CATTTCTCAAAAATCAGTGCTAAGA |
|  |  | Left | GACGCTTTAAAGAGAGATAAAGATGC |
| *P63* | 45 | Right | AATCTGCTGGTCCATGCTGT |
|  |  | Left | GGAAAACAATGCCCAGACTC |
| *Pecam* | 45 | Right | CGACAGGATGGAAATCACAA |
|  |  | Left | CGGTGTTCAGCGAGATCC |
| *Pitx2* | 40 | Right | CCAAAGCCATTCTTGCACA |
|  |  | Left | CCTTACGGAAGCCCGAGT |
| *Sox9* | 66 | Right | TCCACGAAGGGTCTCTTCTC |
|  |  | Left | CAGCAAGACTCTGGGCAAG |
| *Vegfa* | 4 | Right | AGAGGTCTGGTTCCCGAAA |
|  |  | Left | TTAAACGAACGTACTTGCAGATG |
| *Wif1* | 40 | Right | GGCAGACACTGCAATAAGAGG |
|  |  | Left | TTAAGTGAAGGCGTGTGTCG |
| *Wisp1* | 76 | Right | GTGGACATCCAACTACACATCAA |
|  |  | Left | AAGTTCGTGGCCTCCTCTG |
| *Wnt2b* | 16 | Right | CCGGGACCACACTGTCTTT |
|  |  | Left | GCTGACGAGATAGCATAGACGA |
| *Wnt3* | 48 | Right | GATCCAGCCGCACAATCTAC |
|  |  | Left | GCCAAGAGTGTATTCGCATCTA |
| *Wnt3a* | 76 | Right | GAGTGCTCAGAGAGGAGTACTGG |
|  |  | Left | CTTAGTGCTCTGCAGCCTGA |
| *Wnt7a* | 12 | Right | CGCTGGGAGAGCGTACTG |
|  |  | Left | CGATAATCGCATAGGTGAAGG |
| *Wnt7b* | 49 | Right | GCGTCCTCTACGTGAAGCTC |
|  |  | Left | TCTTGTTGCAGATGATGTTGG |
| *Wnt8a* | 75 | Right | ACTGCGGCTGTGACGAGT |
|  |  | Left | CCCGAACTCCACGTTGTC |
| *Wnt16* | 92 | Right | CATGAATCTACACAACAACGAAGC |
|  |  | Left | TTTTCCAGCAGGTTTTCACA |
