# Supplementary material for: Transient Inhibition of FGFR2b-Ligands Signaling Leads to Irreversible Loss of Cellular β-Catenin Organization and Signaling in AER during Mouse Limb Development
Source: PLoS One. 2013 Oct 22;8(10):e76248. doi: 10.1371/journal.pone.0076248 (PMC3805551; doi:10.1371/journal.pone.0076248)
Supplement: Table S2 — Genes up and down regulated in the WNT pathway. (DOCX) [file pone.0076248.s007.docx]

**Table S2. Genes up and down regulated in the WNT pathway**

| **Genes up-regulated** (6 hrs Dox IP vs. 0 hrs E11 forelimbs) | Expression in the limb | | Fold Change  (p-Value) |
| --- | --- | --- | --- |
|  | E11 | E13 |  |
| *Frzb* | Ventral Mesenchyme [1,2] | Pre-chondrogenic and Non-chondrogenic Mesenchyme [1] | 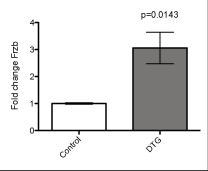 Fold Change: 3.06  p=0.0143 |
| *Pitx2* | Developing Muscle Groups (located at posterior region of limb) [3] | Limb Bud Myoblasts [41] | 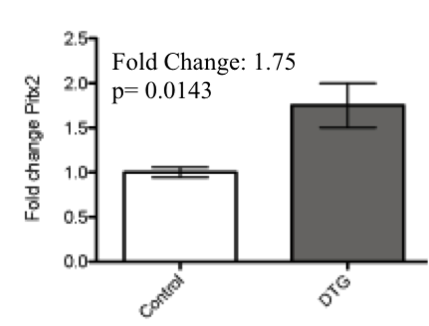 |
| *Wnt8a* | Not Present [1,4] | Not Present [1] | 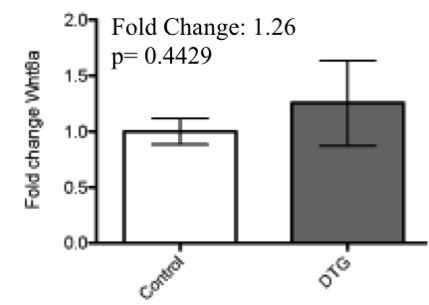 |

| **Genes down-regulated** (6 hrs Dox IP vs. 0 hrs E11 forelimbs) | Expression in the limb | | Fold Change  (p-Value) |
| --- | --- | --- | --- |
|  | E11 | E13 |  |
| *Wnt2b* | Not Present [1,4] | Proximal Interdigital Mesenchyme [1] | 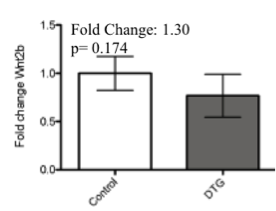 |
| *Wnt3a* | Not Present [1,4] | Not Present [1] | 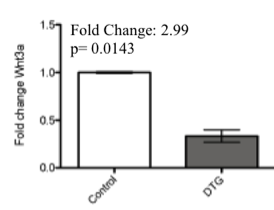 |
| *Wnt3* | Ectoderm (Proximal Epithelium and Handplate Boundary) [1,4] | Ectoderm and Basal Cell Layer [1] | 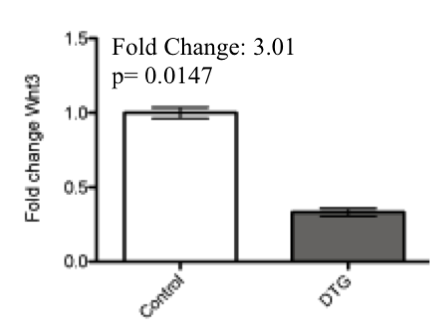 |
| *Wnt7a* | Limb Ectoderm (Ventral in Proximal limb and Dorsal in distal Limb) [1,4] | Not Present [1] | 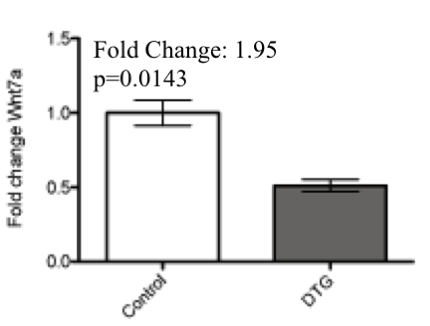 |
| *Wnt7b* | Ventral Ectoderm [1,4] | Basal Cells of the Epidermis [1] | 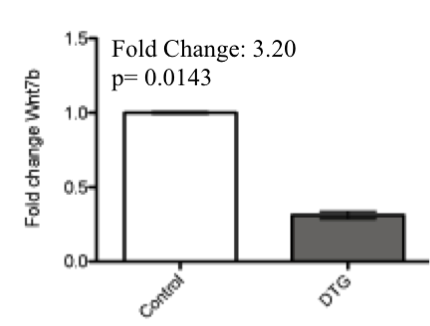 |
| *Wnt16* | Limb Handplate Mesenchyme [1,4] | Carpal Condensations at Metacarpal-Phalangeal Joints [1] | 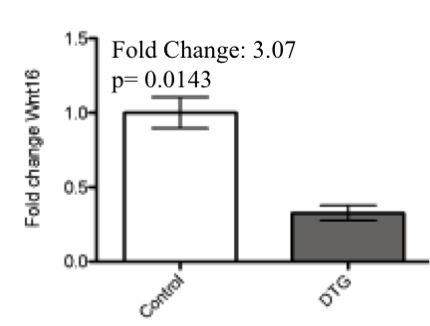 |
| *Fzd4* | 3 Discrete Mesenchymal Patches: posterior distal region of progress zone, and dorsal and ventral patches anterior to midline [4] | Not Reported | 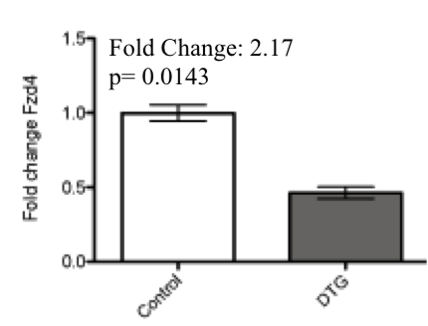 |
| *Fzd8* | Mesenchymal Patches (somewhat complementary to Fzd4) [4] | Not Reported | 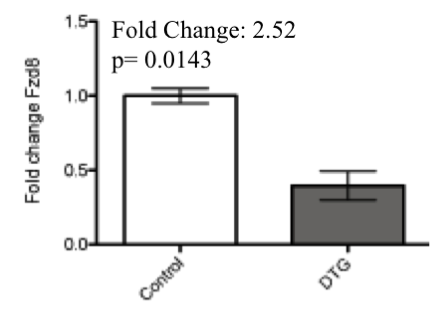 |
| *Fzd9* | Mesenchyme (future digits in distal limb bud and dorsal mesenchme of limb shaft) [4] | Not Reported | 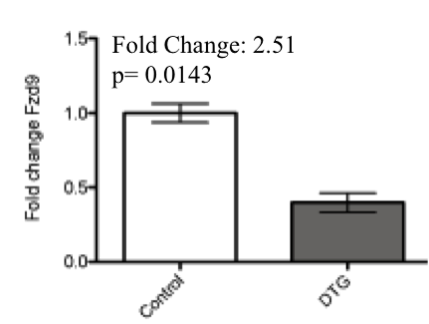 |
| *Wif1* | Proximal Mesenchyme [1] | Distal Mesenchyme [1] | 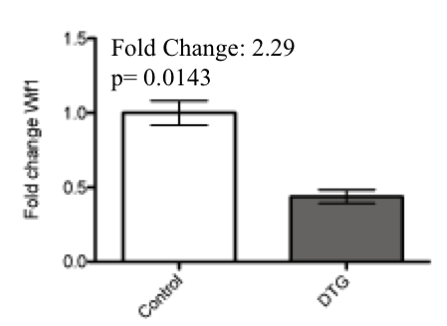 |
| *Wisp1* | Proximal Mesenchyme [1] | Distal Mesenchyme, Interdigital Mesenchyme, Joints [1] | 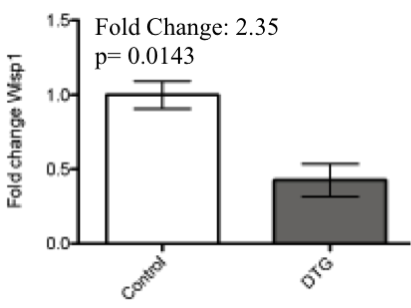 |

1. Witte F, Dokas J, Neundorf F, Mundlos S, and Stricker S. (2009) Comprehensive expression analysis of all Wnt genes and their major secreted antagonists during mouse limb development and cartilage differentiation. Gene Expr Patterns. 9(4):215-223.

2. Ladher RK, Church VL, Allen S, Robson L, Abdelfattah A, et al. (2000) Cloning and expression of the Wnt antagonists Sfrp-2 and Frzb during chick development. Dev Biol. 218(2):183-198.

3. Shih HP, Gross MK, Kioussi C. (2007) [Expression pattern of the homeodomain transcription factor Pitx2 during muscle development.](http://www.ncbi.nlm.nih.gov/pubmed/17166778) Gene Expr Patterns. 7(4):441-451.

4. [Summerhurst K](http://www.ncbi.nlm.nih.gov/pubmed?term=Summerhurst%20K%5BAuthor%5D&cauthor=true&cauthor_uid=18364260), [Stark M](http://www.ncbi.nlm.nih.gov/pubmed?term=Stark%20M%5BAuthor%5D&cauthor=true&cauthor_uid=18364260), [Sharpe J](http://www.ncbi.nlm.nih.gov/pubmed?term=Sharpe%20J%5BAuthor%5D&cauthor=true&cauthor_uid=18364260), [Davidson D](http://www.ncbi.nlm.nih.gov/pubmed?term=Davidson%20D%5BAuthor%5D&cauthor=true&cauthor_uid=18364260), [Murphy P](http://www.ncbi.nlm.nih.gov/pubmed?term=Murphy%20P%5BAuthor%5D&cauthor=true&cauthor_uid=18364260). (2008) 3D representation of Wnt and Frizzled gene expression patterns in the mouse embryo at embryonic day 11.5 (Ts19). Gene Expr Patterns. 8(5):331-348.
